# Supplementary material for: Inter-chromosomal insertions at Xq27.1 associated with retinal dystrophy induce dysregulation of LINC00632 and CDR1as/ciRS-7
Source: Am J Hum Genet. 2025 Jan 31;112(3):523–36. doi: 10.1016/j.ajhg.2025.01.007 (PMC11947168; doi:10.1016/j.ajhg.2025.01.007)
Supplement: Document S1. Figures S1–S9, Tables S2 and S4, and supplemental methods [file mmc1.pdf]

The American Journal of Human Genetics, Volume *112*

Supplemental information

Inter-chromosomal insertions at Xq27.1 associated with retinal dystrophy induce dysregulation of *LINC00632* and *CDR1as/ciRS-7*

Jessica C. Gardner, Katarina Jovanovic, Daniele Ottaviani, Uirá Souto Melo, Joshua Jackson, Rosellina Guarascio, Kalliopi Ziaka, Kwan-Leong Hau, Amelia Lane, Rachel L. Taylor, Niuzheng Chai, Christina Gkertsou, Owen Fernando, Monika Piwecka, Michalis Georgiou, Stefan Mundlos, Graeme C. Black, Anthony T. Moore, Michel Michaelides, Michael E. Cheetham, and Alison J. Hardcastle

Supplemental Figures

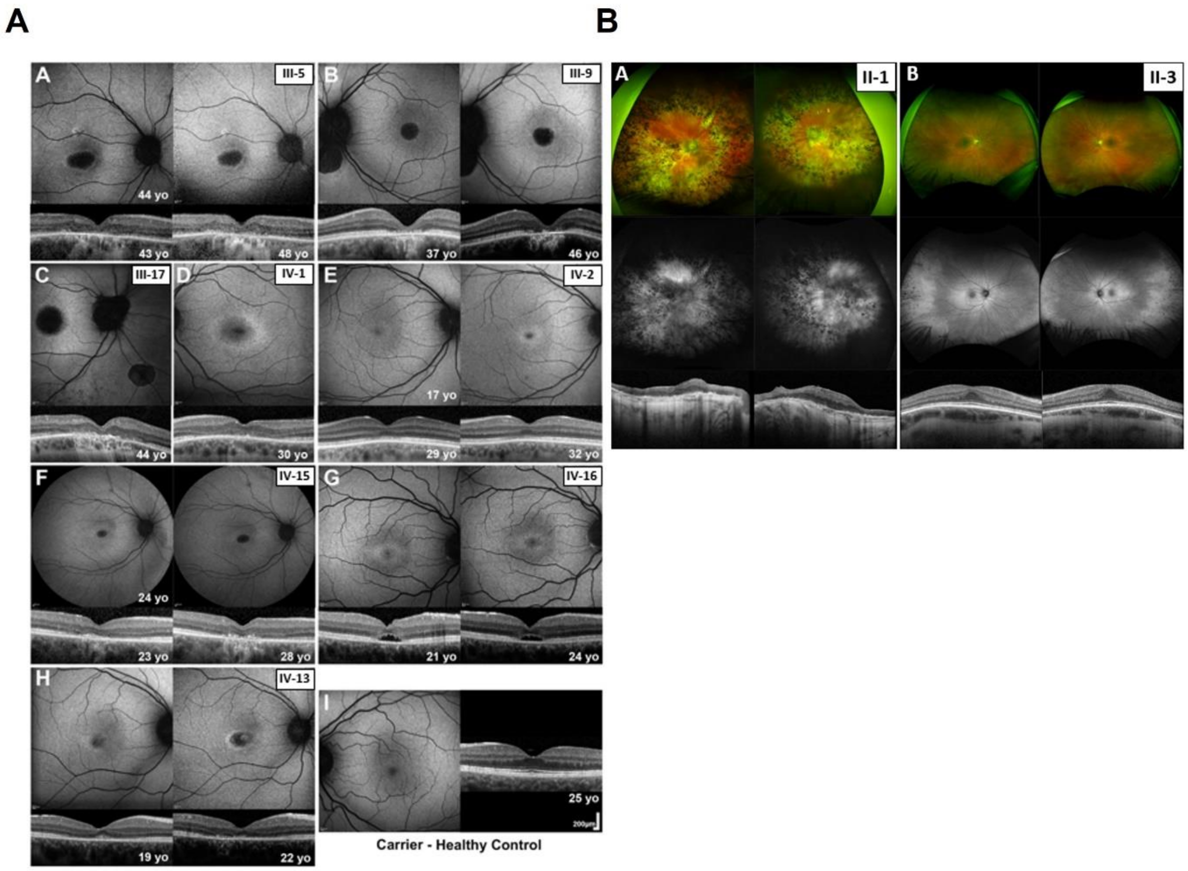

Figure S1: Retinal Imaging

(A) IRDF-1. (A-H) Fundus autofluorescence (FAF) and optical coherence tomography (OCT) of seven individuals with variable degrees of retinal degeneration. (I) FAF and OCT of an asymptomatic carrier as a healthy control. The age of each individual is indicated on the figure. For A, B, E, F, G, H, longitudinal data are presented showing progressive changes. For individuals C and D cross-sectional data are presented. The degeneration followed a cone-dystrophy/cone-rod dystrophy pattern, ranging from (E) a nearly normal FAF pattern and mild ellipsoid zone disruption on OCT to (C) advanced macular atrophy with decrease signal on FAF and atrophic changes on OCT. yo; years old. **(B)** IRDF-2. Top row colour fundus photographs, middle row FAF and bottom row transfoveal OCT of two subjects from IRDF-2. (A) Individual II:1 shows advanced atrophic changes. (B) Individual II:3 has a less severe phenotype with preserved foveal structure, tapetal like reflex, and peripheral degenerative changes.

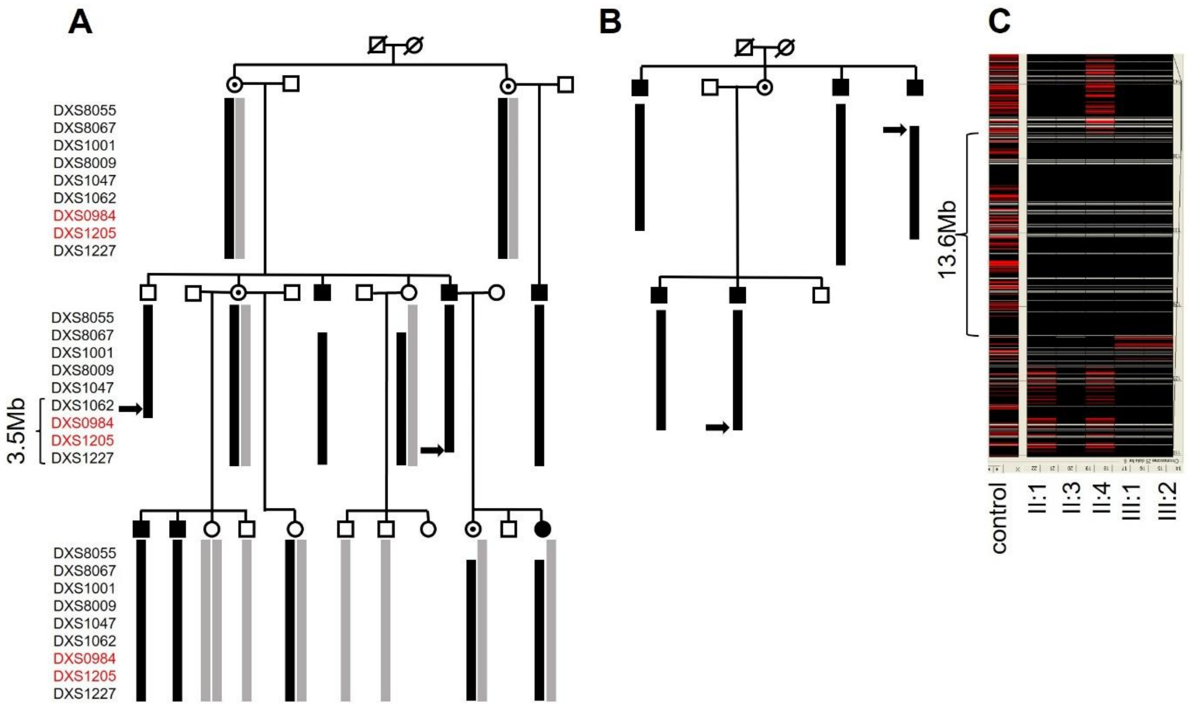

Figure S2: Haplotype analysis

(A) IRDF-1. Black bars indicate chromosome X retinal dystrophy associated haplotypes. Gray bars indicate X chromosome haplotypes that do not segregate with disease. Arrows represent individuals with informative crossovers that define a 3.5Mb retinal dystrophy shared haplotype (chrX:138,221,084-141,714,225 hg38) on Xq26.3-Xq27.2. (markers linked with retinal dystrophy highlighted in red). **(B)** IRDF-2. Black bars indicate chromosome X retinal dystrophy haplotypes in five affected individuals. **(C)** Chromosome X SNP array genotyping data of 5 affected individuals showing a 13.6Mb linked region on Xq25-21.1 in black (chrX:126,856,362-140,533,499 hg38). An unaffected control sample is shown on the left.

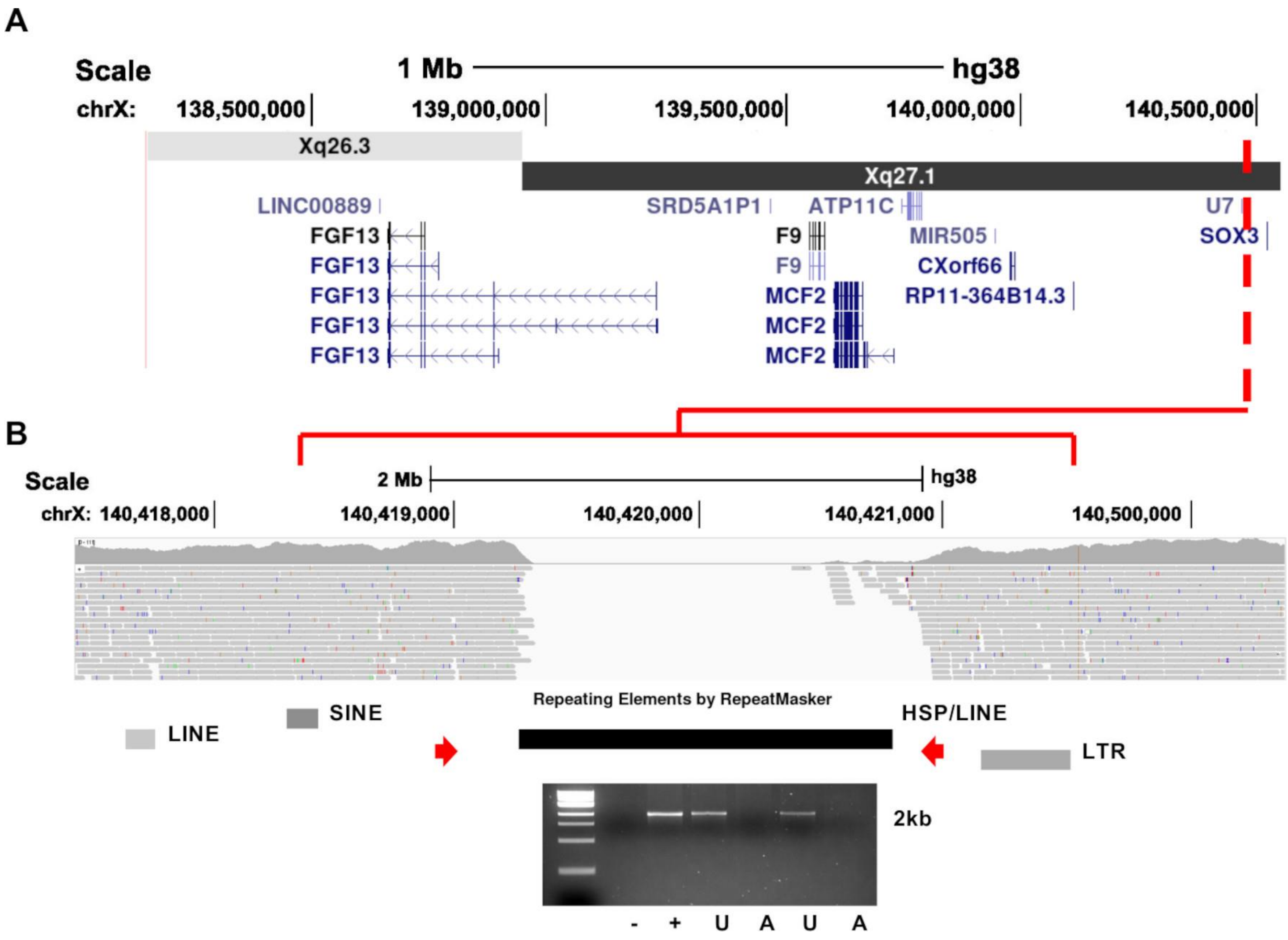

Figure S3: Identification of a genomic region on Xq27.1 poorly covered by NGS

(A) Genomic location (dotted red line) of the sequence gap 80kb downstream of *SOX3* in NGS data for IRDF-1 on Xq27.1. **(B)** Enlarged IGV alignment of NGS gap across a human specific palindrome (HSP) on Xq27.1 (black bar) flanked by an upstream short interspersed nuclear element (SINE) and a downstream long tandem repeat (LTR). Agarose gel (bottom) showing PCR amplification across the gap with primers flanking the HSP (arrows). A 2kb amplicon is present in control (+) and unaffected (U) IRDF-1 individuals, but not in affected individuals (A), indicating a possible SV.

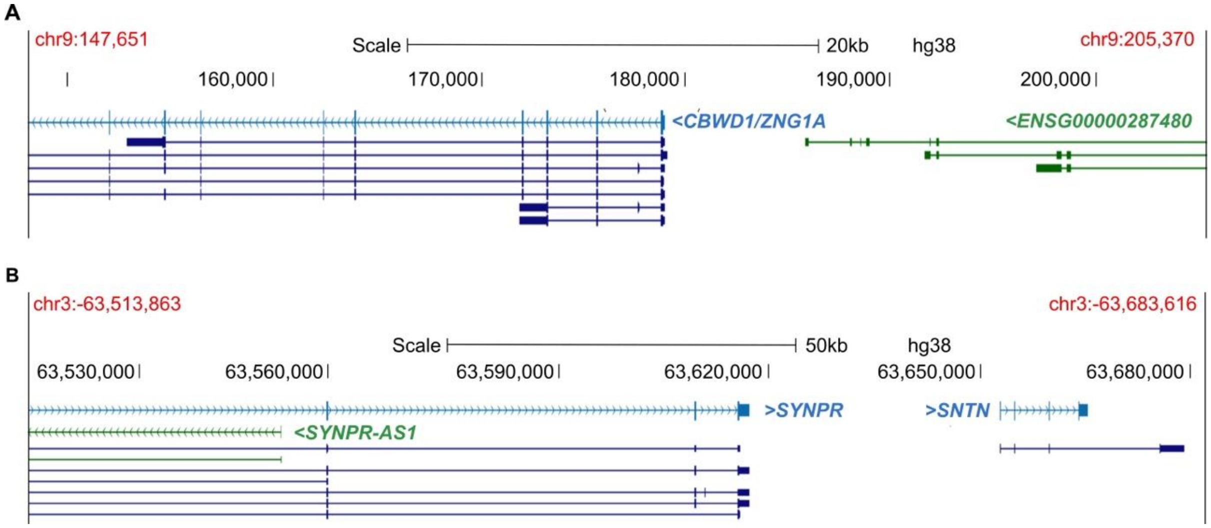

Figure S4: Genes and transcripts within the 9q24.3 and 3p14.2 inter-chromosomal insertions
(A) A 58kb 9p24.3 inter-chromosomal insertion identified in family IRDF-1 contains exons 1-10 and the upstream region of the *CBWD1/ZNG1A* gene and terminal exons of a non-coding RNA. **(B)** A 169kb inverted inter-chromosomal insertion identified in family IRDF-2 contains the *SNTN* gene, three terminal exons of *SYNPR* and the upstream region and first exon of *SYNPR-AS1*. Breakpoint (BP) co-ordinates (in red) for the insertions are also shown.

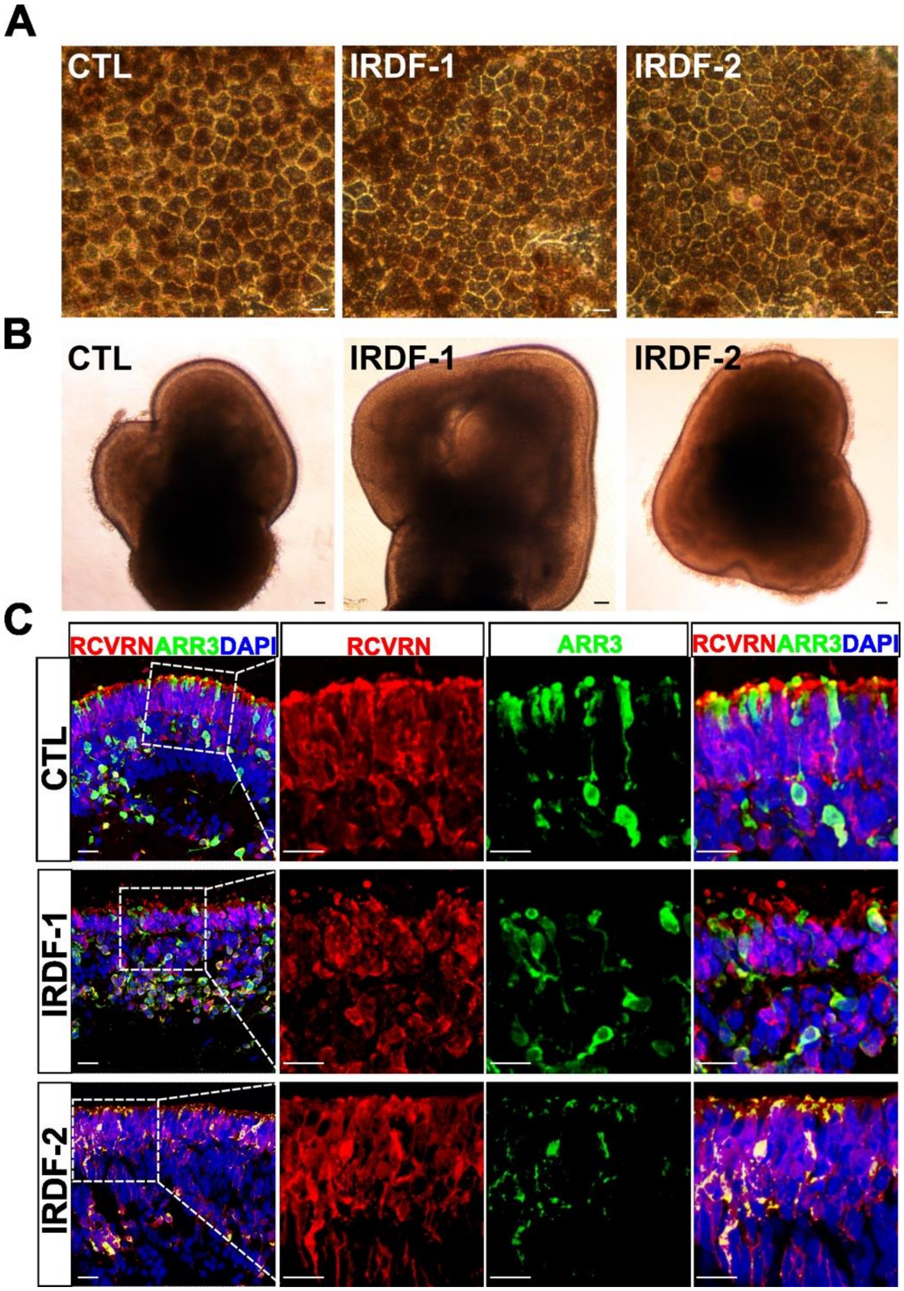

Figure S5: Characterisation of IRDF-1, IRDF-2 and control iPSC-derived RPE and retinal organoids

(A) Brightfield microscopy images of RPE derived from control (CTL), IRDF-1 and IRDF-2 derived iPSC, as indicated. Note the hexagonal shape and pigmentation characteristic of RPE. Scale bar 20 µm**. (B)** Brightfield microscopy images of representative retinal organoids derived from CTL, IRDF-1 and IRDF-2 iPSC, as indicated. Scale bar 50 µm. **(C)** Immunohistochemistry of 10 µm cryosections of D150 retinal organoids from CTL, IRDF-1 and IRDF-2 iPSC, as indicated, stained with primary antibodies against photoreceptor markers recoverin (RCVRN, red), cone arrestin (ARR3, green) showing rod and cone photoreceptor differentiation. Nuclei are stained with DAPI (blue), note the discrete outer nuclear layer of photoreceptors confirming retinal organoid lamination. Scale bar 20 µm.

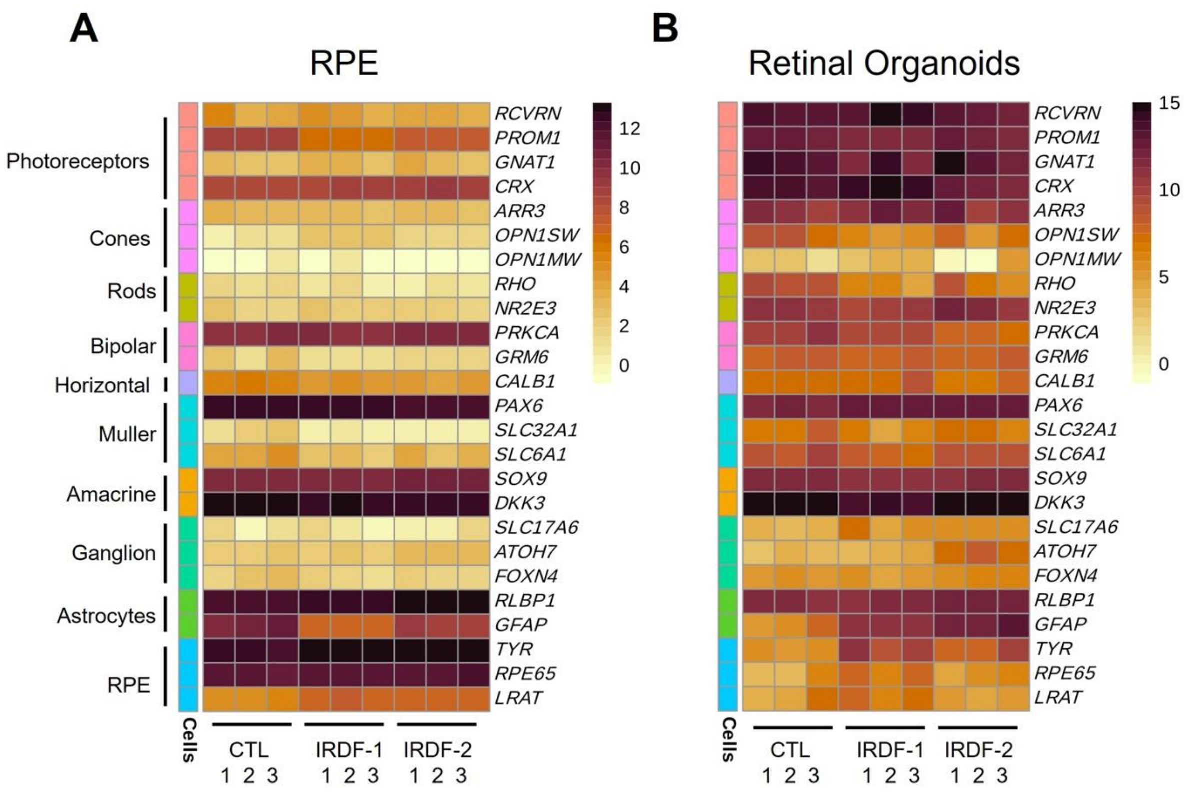

Figure S6: Heat maps of gene expression profiles generated from RNA-seq data from RPE and retinal organoids

(A) Gene profiles of RPE samples (3 samples per line). **(B)** Marker gene profiles of D150 ROs retinal cell types including photoreceptor-specific genes. Comparison between control (CTL) and IRDF-1 and IRDF-2 (3 ROs per line). Both tissue types differentiated successfully and expressed tissue specific genes.

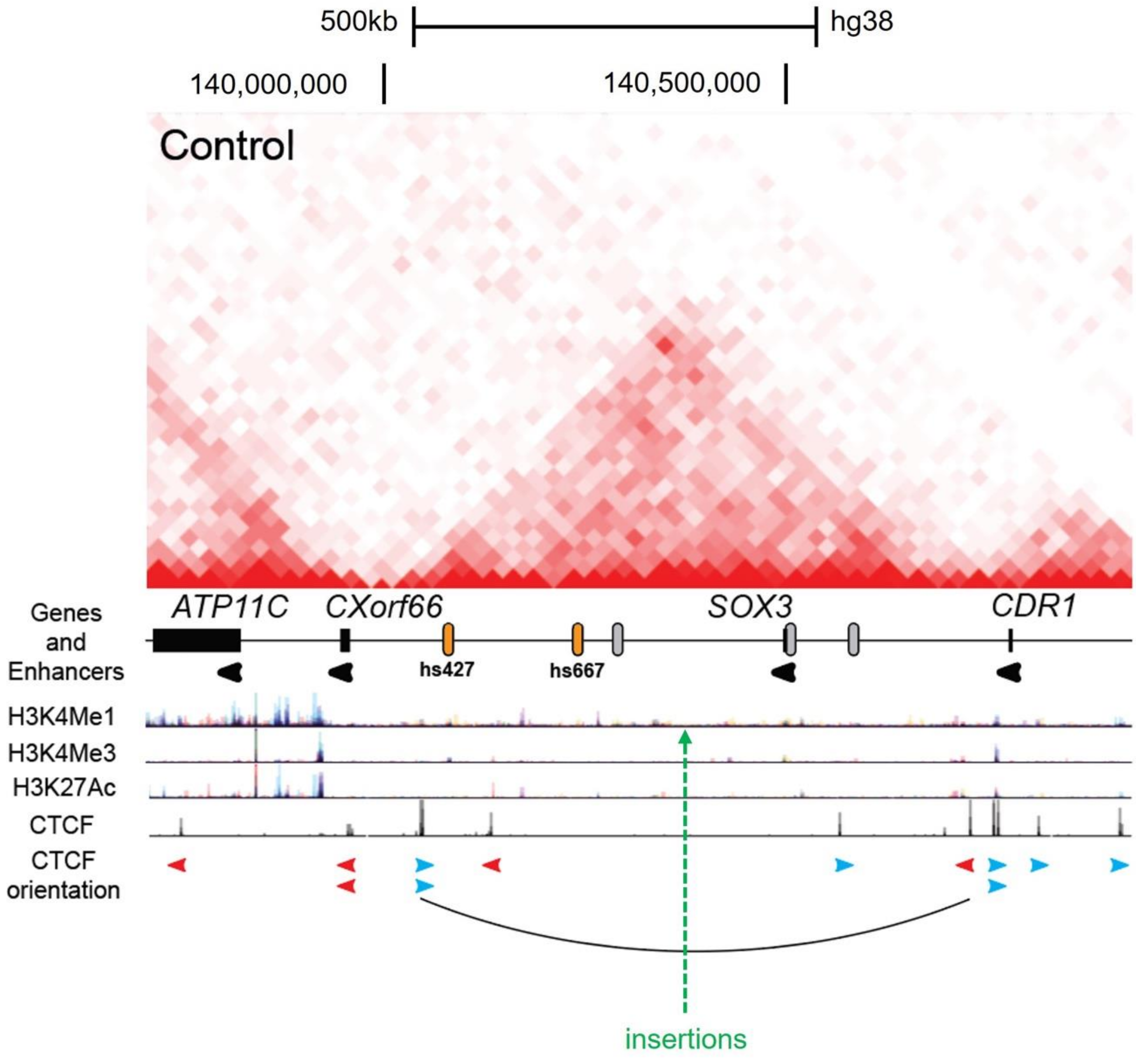

Figure S7: CTCF-associated *SOX3* TAD boundaries

CTCFs at the *SOX3* TAD boundaries are arranged in the classical convergent orientation. The position of the insertions is indicated by the green arrow.

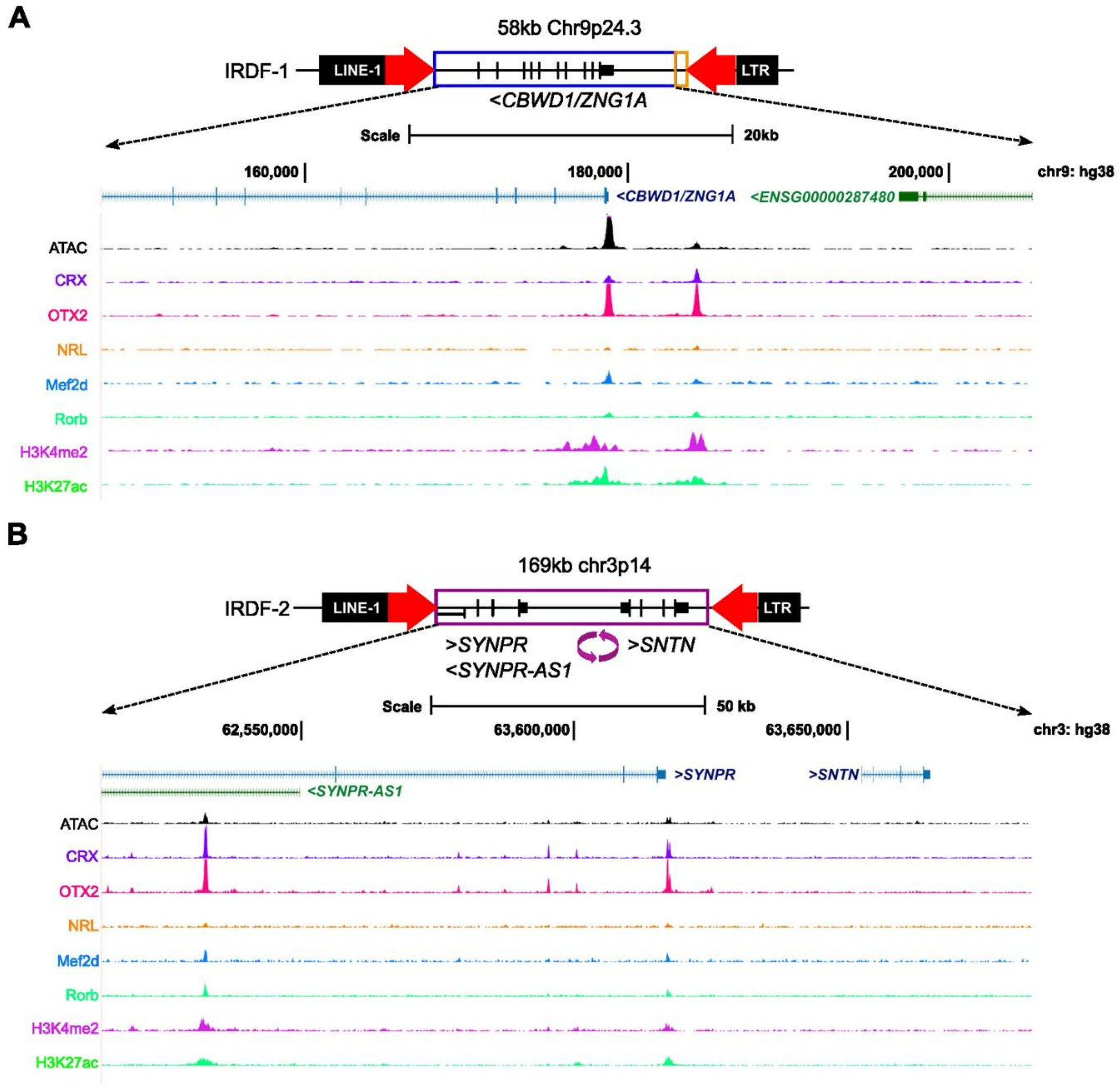

Figure S8. Active regulatory elements correspond with photoreceptor specific enhancers within the ICIs

Multiomic data aligned to **(A)** the 9p24.3 insertion in IRDF-1 and **(B)** the 3p14.2 insertion in IRDF-2 reveals open chromatin (ATAC, H3K27ac) and signatures of active retina specific enhancers (*OTX2*, *CRX*) within both insertions.

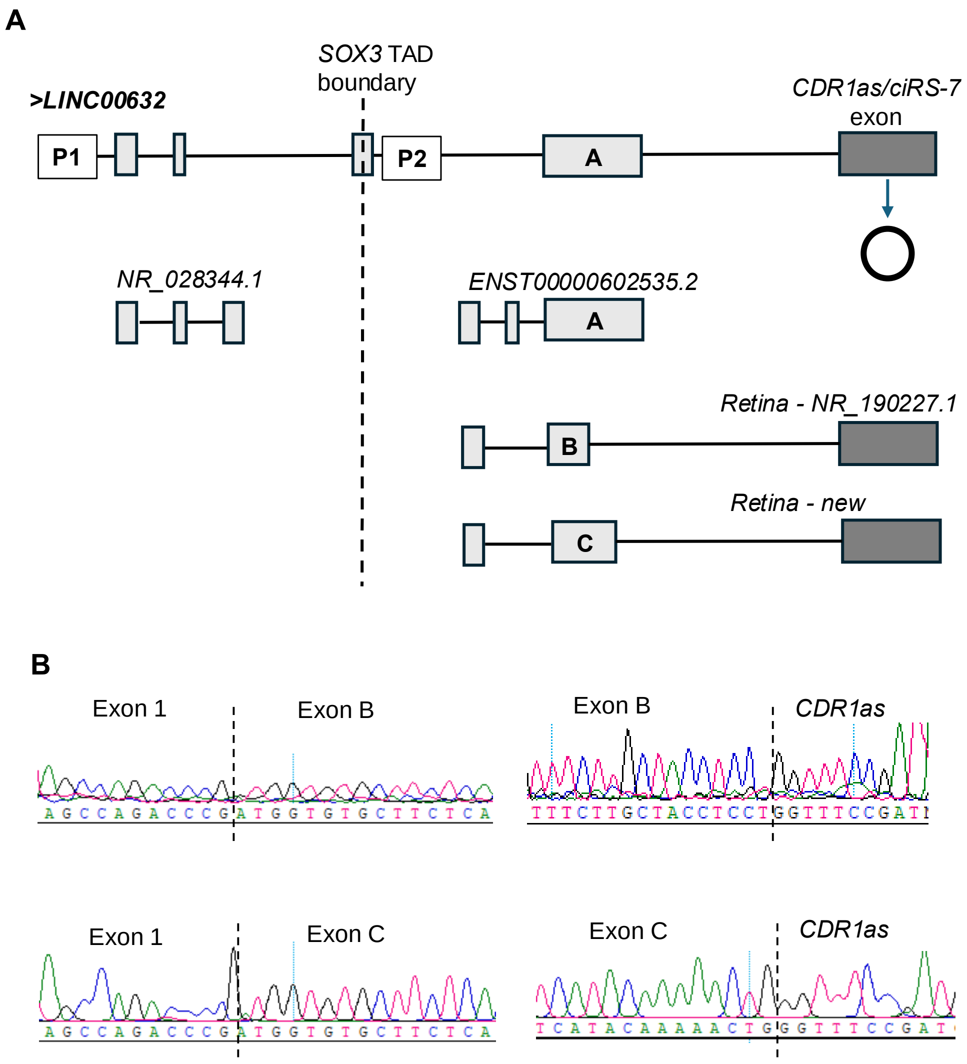

Figure S9: Expression of *CDR1as/ciRS-7* in retina is driven by two promoters on different sides of the *SOX3* TAD boundary

(A) Schematic showing *LINC00632* transcripts and promoters, P1 and P2, relative to the position of the distal *SOX3* TAD boundary. **(B)** Sequence chromatograms of two shorter transcripts with a terminal *CDR1as* exon detected by RT-PCR in D150 control retinal organoids showing the splice junctions. Both transcripts are associated with P2 and lie outside of the *SOX3* TAD boundary. Note, not all putative *LINC00632* transcripts in ENSEMBL or UCSC are depicted on this figure.

Supplemental Tables

Table S2: Primers used for the identification and characterisation of SVs and to characterise linear *LINC00632* transcripts

Target	Primer Name	Primer sequence (5’-3’)	Position hg38
**Human Specific Palindrome**	HSPF	CATTGAAATCAACCACCCAG	chrX:140,418,915
	HSPR	AACATTTCCAATCTCCCCAAC	chrX:140,420,906
**DNA Walking**	R-TSP1	GGTCCAATGCTTTGTAGTG	chrX:-140,421,014
	R-TSP2	ACCAGAGTTCTCTGTGCATTG	chrX:-140,420,964
	R-TSP3	AACATTTCCAATCTCCCCAAC	chrX:-140,420,906
	F-TSP1	GTAGTGTGATGCCTCCAGC	chrX:140,420,580
	F-TSP2	ATTGACTTGGCGATGCG	chrX:140,420,618
	F-TSP3	GAAGAAAGTCATTGGGGTTCAGC	chrX:140,420,686
**IRDF-1 BP**	Chr9R1	AAATATGCTCAGTAGCGGGG	chr9:-147723
***LINC00632* (P2) linear transcripts with promoter outside SOX3 TAD**	Forward<br>Reverse	AGCCAGACCCGATGGTGTG<br>AAGACCCGGAGTTGTTGGAA	Exon1/2<br>CDR1as
***LINC00632*/NR_19022.1 nested from *LINC00632* (P2)**	Forward<br>Reverse	AGCCAGACCCGATGGTGTG<br>ATCGGAAACCAGGAGGTAGC	Exon1/2<br>Ex2A/CDR1as
***LINC00632*/NEW nested from *LINC00632* (P2)**	Forward<br>Reverse	AGCCAGACCCGATGGTGTG<br>GTGCCATCGGAAACCCAG	Exon1/2<br>Ex2B/CDR1as

Table S4: RT-qPCR primers used to validate differential expression of linear and circular isoforms of *LINC00632*

Target	Primer	Primer sequence (5'-3')
ACTIN	Forward	CCAACCGCGAGAAGATGA
	Reverse	CCAGAGGCGTACAGGGATAG
GAPDH	Forward	CCCCACCACACTGAATCTCC
	Reverse	GGTACTTTATTGATGGTACATGACAAG
LINC00632-NR_028344.1	Forward	AGACAGCATGCCACTGGAAA
	Reverse	CTGCGGACACAAGTCTGCTT
CDR1as/ciRS-7	Forward	CGTCTCCAGTGTGCTGATCT
	Reverse	AAGACCCGGAGTTGTTGGAA

Supplemental Methods

Genotyping

The IRDF-1 locus was initially established using polymorphic markers on ChrX that were genotyped in 17 individuals (ABI PRISM® linkage mapping set version 2.5, Thermo Fisher Scientific, UK; and additional microsatellite markers - primers and conditions available upon request). The locus was subsequently refined by SNP genotyping following genome sequence analysis of additional individuals (two affected IV-15, IV-16 and unaffected III-1). SNVs were validated using PCR amplification and Sanger Sequencing (primers and conditions available on request). The IRDF-2 locus was identified using SNP genotyping in 5 affected individuals. Genome-Wide SNP analysis was carried out using the Affymetrix Genome-Wide SNP6.0 microarray. Genotypes were generated using the Birdseed V2 algorithm with a confidence threshold of 0.01 and copy number data was generated using the SNP 6.0 CN/LOH Algorithm both within the Affymetrix Genotyping console. Autozygosity analysis was carried out using AutoSNPa (http://dna.leeds.ac.uk/autosnpa/). Copy number results were analysed using the Affymetrix Chromosome Analysis Suite.

Sanger sequencing of candidate genes

In IRDF-1 and IRDF-2 the coding regions and intron/exon boundaries of all candidate genes (including reference and predicted genes in GRCh37/hg19) within the linked loci were amplified by PCR and bidirectionally sequenced under standard conditions using the BigDye Terminator v 3.1 kit (Life Technologies).

Array Comparative Genome Hybridisation (CGH)

In IRDF-1, genomic DNA of an affected male (III-5) was analysed for copy number variations (CNVs) of the X chromosome by array CGH (Roche, NimbleGen, Madison, USA) using the NimbleGen custom microarray services facility (NimbleGen Systems of Iceland, LLC, Reykjavik, Iceland). DNA was labelled with Cy3 or Cy5 using a NimbleGen Dual Color DNA labelling kit and co-hybridized to the arrays (NimbleGen CGH Services: Guide to your CGH data v5p1). Design of the array was based on GRCh36/hg18.

Exome and genome sequencing

WES was performed for one affected individual of IRDF-1 (IV-1) and IRDF-2 (III-1). Library preparation was performed using the Agilent Human Exome V4 capture kit and run on an Illumina HiSeq2500TM system by Otogenetics Corporation (Atlanta, USA). After quality control, reads were aligned to the GRCh37 sequence with BWA v.0.78 and variants were called using GATK HaplotypeCaller V.3.3. CNVs and SVs were analysed from WES data using ExomeDepth, Manta Structural Variant Caller, Canvas Copy Number Variant Caller and Control-FREEC.

In IRDF-1 targeted genome sequencing of the linked region on chrX was performed in one affected male (III-5) and two unrelated male control samples. Genomic DNA was independently captured using a Sequence Capture 2.1M Custom Array (Roche, NimbleGen) to enrich five target regions of interest (chrX: 8,353,912–19,064,721; 37,980,770–38,109,739; 46,569,929–46,638,096; 135,964,421–144,028,731; 153,000,017–153,246,396; GRCh36/hg18). 100bp paired-end sequencing was performed on an Illumina Genome Analyzer II system (Source Bioscience Geneservice). Initial bioinformatics analysis was performed using CASAVA (Illumina). Advanced bioinformatic analysis was performed using the SAMtools software toolkit (SNP and DIP detection). SNPs were called with the MAQ alignment and downstream analysis tools (Source Bioscience Geneservice). Data were then interrogated using a variety of bioinformatics tools.

Short read WGS was performed using Macrogen Inc. (Seoul, Korea) on Illumina HiSeqX machines using a 2x 100bp paired end module with a minimum median coverage per genome of 30-fold. For IRDF-1, two affected individuals (IV-15, IV-16) and one unaffected (III-1) were sequenced. For IRDF-2, one affected individual (III-1) was sequenced. After quality control, reads were aligned with BWA to map reads against reference genomes (GRCh37/hg37 and GRCh38/hg38) and GATK HaplotypeCaller (V.3.3) for variant calling. Variants were filtered using gnomAD MAF<0.001, validated with IGV software (V.2.4), and selected for purposes of refining the locus. CNVs and SVs were analysed using Canvas Copy Number Variant Caller (Illumina) and Manta Structural Variant Caller.

Identification and validation of structural variants

A dark or camouflaged genome sequence gap within the IRDF-1 locus was investigated using long range PCR amplification, with sequence specific primers HSPF and HSPR (Table S2, PCR conditions available on request). The 1.8kb sequence gap spanned a complex intergenic repeat on Xq27.1, comprising a 180bp human specific palindrome, flanked by long tandem repeat (LTR) and a long intergenic nuclear element (LINE/L1PA3).

A genome walking strategy to identify unknown sequence within the gap was performed using genomic DNA from an affected male (IV-1) in IRDF-1 and was subsequently repeated in IRDF-2 (III-1). The DNA Walking SpeedUp™ premix kit (Seegene, Inc. Seoul, Korea) was used according to the manufacturer's instructions. gDNA from an affected individual was amplified using DW-ACP™ primers from the kit paired with a set of nested target-specific (TSP) primers designed against the centromeric (TSPF1-3) and telomeric (TSPR1-3) flanks of the palindrome (Table S2). An initial DNA walking PCR, using primers DW-ACP™ and TSP1, was employed to amplify unknown sequence, followed by two rounds of PCR using DW primers and nested TSP2-3 primers with products from the previous PCR. The amplification products were separated by agarose gel electrophoresis and extracted using a Qiaquick gel extraction DNA kit (Qiagen,UK) before Sanger sequencing. Breakpoint PCR was performed to validate the proximal chrX breakpoint of IRDF-1 using primers HSPF and Chr9R1 (Table S2). PCR conditions are available on request.

Interrogation of the genomic region

To explore the regulatory landscape of the chrX region and inter-chromosomal insertion regions on Chr9p and Chr3p we interrogated the UCSC encode data sets and the integrated retinal multi-omic data base RegRet.[1]

Cell culture

Fibroblasts were cultured in Dulbecco's Modified Eagle Medium (DMEM; Thermo Fisher Scientific) supplemented with 15% fetal bovine serum (FBS; Thermo Fisher Scientific), 1% L-glutamine (Thermo Fisher Scientific), and 1% penicillin-streptomycin (Thermo Fisher Scientific) before reprogramming to iPSC.

Preparation of Hi-C libraries

Hi-C libraries were processed as described previously.[2] In brief, ~1 million cells were fixed in 2% formaldehyde, lysed, and digested overnight with DpnII enzyme (New England BioLabs, R0543). Digested DNA ends were marked with biotin-14-dATP (Thermo Fisher Scientific, 19524016) and ligated overnight using T4 DNA ligase (New England BioLabs, M0202). Formaldehyde crosslinking was reversed by incubation in 5 M NaCl for 2 h at 68°C, followed by ethanol precipitation. Covaris (S-Series 220) was used to shear the DNA to fragments of 300–600bp for library preparation, and biotin-filled DNA fragments were pulled down using Dynabeads MyOne Streptavidin T1 beads (Thermo Fisher Scientific, 65602). The DNA ends were subsequently repaired using T4 DNA polymerase and the Klenow fragment of DNA polymerase I (New England BioLabs, M0203 and M0210) and phosphorylated with T4 Polynucleotide Kinase NK (New England BioLabs, M0201). The DNA was further prepared for sequencing by ligating adaptors to the DNA fragments, using the NEBNext Multiplex Oligos for Illumina kit (New England BioLabs, E7335 and E7500). Indexes were added via PCR amplification (4–8 cycles) using the NEBNext Ultra II Q5 Master Mix (New England BioLabs, M0544). PCR purification and size selection were carried out using Agencourt AMPure XP beads (Beckman Coulter, A63881). Libraries were deep sequenced (~240 million fragments for fibroblasts and 320 million fragments for retinal organoids) in a 75bp paired-end run on a HiSeq4000 (Illumina). For each line, the Hi-C library was created by pooling a total of four technical replicates to ensure higher complexity of the sequencing library.

References

1. Van de Sompele, S., Small, K.W., Cicekdal, M.B., Soriano, V.L., D’haene, E., Shaya, F.S., Agemy, S., Van der Snickt, T., Rey, A.D., Rosseel, T., et al. (2022). Multi-omics approach dissects cis-regulatory mechanisms underlying North Carolina macular dystrophy, a retinal enhanceropathy. Am J Hum Genet *109*, 2029–2048. https://doi.org/10.1016/j.ajhg.2022.09.013.
2. Melo, U.S., Schöpflin, R., Acuna-Hidalgo, R., Mensah, M.A., Fischer-Zirnsak, B., Holtgrewe, M., Klever, M.K., Türkmen, S., Heinrich, V., Pluym, I.D., et al. (2020). Hi-C Identifies Complex Genomic Rearrangements and TAD-Shuffling in Developmental Diseases. Am J Hum Genet *106*, 872–884. https://doi.org/10.1016/J.AJHG.2020.04.016.
