# Supplementary material for: Inter-chromosomal insertions at Xq27.1 associated with retinal dystrophy induce dysregulation of LINC00632 and CDR1as/ciRS-7
Source: Am J Hum Genet. 2025 Jan 31;112(3):523–36. doi: 10.1016/j.ajhg.2025.01.007 (PMC11947168; doi:10.1016/j.ajhg.2025.01.007)
Supplement: Document S2. Article plus supplemental information [file mmc5.pdf]

Inter-chromosomal insertions at Xq27.1 associated with retinal dystrophy induce dysregulation of *LINC00632* and *CDR1as/ciRS-7*

Graphical abstract

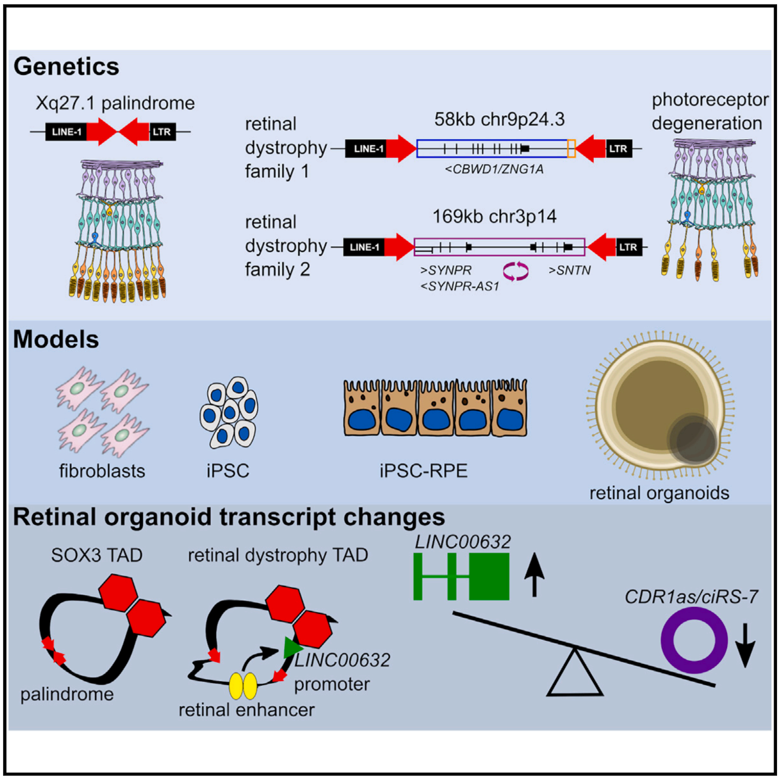

Authors

Jessica C. Gardner, Katarina Jovanovic, Daniele Ottaviani, ..., Michel Michaelides, Michael E. Cheetham, Alison J. Hardcastle

Correspondence

jessica.gardner@ucl.ac.uk (J.C.G.),
a.hardcastle@ucl.ac.uk (A.J.H.)

Inter-chromosomal insertions within a palindrome on Xq27 are associated with rare X-linked diseases, but how they cause disease is unknown. We describe two retinal dystrophy families with different insertions. iPSC-derived models revealed tissue-specific dysregulation of *LINC00632* and its circular isoform, *CDR1as/ciRS-7*, driven by retinal enhancers within the insertions.

Gardner et al., 2025, The American Journal of Human Genetics *112*, 523–536
March 6, 2025 © 2025 The Authors. Published by Elsevier Inc. on behalf of American Society of Human Genetics.
https://doi.org/10.1016/j.ajhg.2025.01.007

Inter-chromosomal insertions at Xq27.1 associated with retinal dystrophy induce dysregulation of *LINC00632* and *CDR1as/ciRS-7*

Jessica C. Gardner,[1,*] Katarina Jovanovic,[1] Daniele Ottaviani,[1,2] Uirá Souto Melo,[3] Joshua Jackson,[1] Rosellina Guarascio,[1] Kalliopi Ziaka,[1] Kwan-Leong Hau,[1] Amelia Lane,[1] Rachel L. Taylor,[4] Niuzheng Chai,[1] Christina Gkertsou,[1] Owen Fernando,[1] Monika Piwecka,[5] Michalis Georgiou,[1,6] Stefan Mundlos,[3,7] Graeme C. Black,[4,8] Anthony T. Moore,[1,6] Michel Michaelides,[1,6] Michael E. Cheetham,[1] and Alison J. Hardcastle[1,*]

Summary

In two unrelated families with X-linked inherited retinal dystrophy, identification of the causative variants was elusive. Interrogation of the next-generation sequencing (NGS) data revealed a "dark" intergenic region on Xq27.1 with poor coverage. Long-range PCR and DNA walking across this region revealed different inter-chromosomal insertions into the human-specific palindrome on Xq27.1: a 58 kb insertion of 9p24.3 [der(X)dir ins(X;9)(q27.1;p24.3)] in family 1 and a 169 kb insertion of 3p14.2 [der(X)inv ins(X;3)(q27.1;p14.2)] in family 2. To explore the functional consequence of these structural variants in genomic and cellular contexts, induced pluripotent stem cells were derived from affected and control fibroblasts and differentiated to retinal organoids (ROs) and retinal pigment epithelium. Transcriptional dysregulation was evaluated using RNA sequencing (RNA-seq) and RT-qPCR. A downstream long non-coding RNA, *LINC00632* (Xq27.1), was upregulated in ROs from both families compared to control samples. In contrast, the circular RNA *CDR1as/ciRS-7* (circular RNA sponge for *miR-7*), spliced from linear *LINC00632*, was downregulated. To investigate this tissue-specific dysregulation, we interrogated the landscape of the locus using Hi-C and cleavage under targets and tagmentation sequencing (CUT&Tag). This revealed active retinal enhancers within the insertions within a topologically associated domain that also contained the upstream promoter of *LINC00632*, permitting ectopic contact. Furthermore, *CDR1as/ciRS-7* acts as a "sponge" for *miR-7*, and target genes of *miR-7* were also dysregulated in ROs derived from both families. We describe a new genomic mechanism for retinal dystrophy, and our data support a convergent tissue-specific mechanism of altered regulation of *LINC00632* and *CDR1as/ciRS-7* as a consequence of the insertions within the palindrome on Xq27.1.

Introduction

Inherited retinal dystrophies are a group of clinically and genetically heterogeneous disorders characterized by visual disability consequent upon dysfunction and, often, progressive degeneration of rod and/or cone photoreceptors or retinal pigment epithelium (RPE). Over 315 associated genes and loci are currently described (RetNet database: https://sph.uth.edu/RetNet/); nevertheless, for a significant number of genetically tested individuals, the pathogenic variant remains unidentified. This is likely attributable to non-coding variants, structural rearrangements, and variants in elusive genes.[1–6]

Structural variants (SVs) (rearrangements of >50 bp) are a common form of genetic variation in the human genome, with an average of >25,000 SVs per genome.[7–10] SVs can delete, multiply, or relocate large segments of DNA and are more likely to have a significant functional impact than single-nucleotide variants (SNVs) in the non-coding genome.[11–13] SVs are difficult to detect and challenging to interpret and validate due to shortcomings in next-generation sequencing (NGS) and a lack of appropriate cellular and animal models of disease.[14] Depending on the genomic location, structure (either simple deletion, duplication, inversion, translocation, or a complex combination of rearrangements), and precise location of breakpoints, SVs can cause pathogenic effects by altering gene dosage, positioning, splicing, regulation, 3D chromatin organization, or through a combination of effects.[8,15–17] SVs are increasingly being implicated as the genomic cause of disease. SVs affecting non-coding regulatory regions, or motifs involved in the maintenance of 3D chromatin structure, have been implicated in neurodevelopmental disorders, including autism spectrum disorder, intellectual disability, schizophrenia, and developmental delay.[11,18–22] Complex SVs on chromosome 17 have been associated with autosomal dominant retinitis pigmentosa (RP17 [MIM: 600852]), as a result of disruption of the 3D chromatin architecture of the locus

[1]UCL Institute of Ophthalmology, University College London, London, UK; [2]Department of Biology, University of Padua, Padua, Italy; [3]Max Planck Institute for Molecular Genetics, RG Development & Disease, Berlin, Germany; [4]Division of Evolution, Infection and Genomics, School of Biological Sciences, Faculty of Biology, Medicine and Health, University of Manchester, Manchester, UK; [5]Department of Non-coding RNAs, Institute of Bioorganic Chemistry, Polish Academy of Sciences, Poznan, Poland; [6]Moorfields Eye Hospital NHS Foundation Trust, London EC1V 2PD, UK; [7]Institute for Medical and Human Genetics, Charité Universitätsmedizin, Berlin, Germany; [8]Manchester Centre for Genomic Medicine, Saint Mary's Hospital, Manchester University NHS Foundation Trust, Manchester, UK
*Correspondence: jessica.gardner@ucl.ac.uk (J.C.G.), a.hardcastle@ucl.ac.uk (A.J.H.)
https://doi.org/10.1016/j.ajhg.2025.01.007.
© 2025 The Authors. Published by Elsevier Inc. on behalf of American Society of Human Genetics.
This is an open access article under the CC BY license (http://creativecommons.org/licenses/by/4.0/).

and the formation of new topologically associated domains (neoTADs) that permit ectopic enhancer-gene contacts leading to the dysregulation of gene expression in the region.[23]

We investigated two retinal dystrophy families in which a genetic diagnosis had not been established. We report mapping a locus on Xq25–q27.2 and subsequent identification and characterization of two different inter-chromosomal insertions within a repeat-rich intergenic region at Xq27.1. We used induced pluripotent stem cells (iPSCs) from affected individuals, differentiated into RPE and 3D retinal organoids (ROs), to explore the mechanism of disease in genomic and cellular contexts.

Material and methods

Ethics statement

The study adhered to the World Medical Association Declaration of Helsinki and was approved by the institutional review boards (IRBs) of Moorfields Eye Hospital, University College London, UK (Genetic Study of Inherited Eye Disease; research ethics committee [REC] reference 12/LO/0141), and Manchester Hospital NHS Trust. Consent was obtained from participants or their legal representative for all clinical and molecular studies in this report.

Clinical analysis

The index family, inherited retinal dystrophy (IRD) family 1 (IRDF-1), was recruited at Moorfields Eye Hospital, London, and IRDF-2 was recruited at Manchester Hospital NHS Trust. Participating affected and unaffected individuals underwent detailed ophthalmological investigation including visual acuity and field testing, fundus ophthalmoscopy and imaging, spectral domain optical coherence tomography (SD-OCT), and full-field electroretinography (ERG) studies.

Molecular genetic analysis

Genomic DNA was isolated from peripheral blood for genomic mapping and sequencing. Following the exclusion of known X-linked retinal dystrophy genes (*RPGR* and *RP2* [MIM: 312610 and MIM: 300757]), DNA haplotypes were constructed from genotyped microsatellite markers (IRDF-1), genome-wide single-nucleotide polymorphism (SNP) microarrays (IRDF-2), and SNVs (IRDF-1 and IRDF-2) to define and refine the loci (supplemental methods). Further interrogation of the X-linked loci included Sanger sequencing of candidate genes within the loci, array comparative genome hybridization (CGH), exome sequencing, and short-read genome sequencing (supplemental methods). Sequence data were aligned to Human Reference Genome builds hg18 (chromosome X targeted genome sequence), hg19 (Sanger and whole-exome sequencing [WES]), and hg38 (whole-genome sequencing [WGS]). Validation and segregation of selected rare candidate variants (minor-allele frequency [MAF] < 0.001) was performed by PCR and Sanger sequencing following standard protocols. Genome data were further manually inspected using the Integrative Genome Viewer (IGV) software (v.2.4) (http://software.broadinstitute.org/software/igv/).

Identification and validation of SVs

A 1.8 kb dark region of a repetitive genome sequence within the linked locus on Xq27.1 of IRDF-1, and subsequently IRDF-2, was investigated using long-range PCR amplification, DNA walking, IGV visualization of WGS split reads, and the UCSC blat tool (https://genome.ucsc.edu/cgi-bin/hgBlat) (supplemental methods). SV breakpoints identified by DNA walking or breakpoint PCR were Sanger sequenced. Primer sequences are listed in Table S2. SV breakpoints were reviewed for the presence of repeat elements and microhomology.

Interrogation of the genomic region

The epigenomic landscape of the Xq27.1 region and the two insertions were explored using publicly available chromatin and genome regulation datasets using the UCSC Genome Browser and data derived from retinas.[24,25]

Reprogramming fibroblasts to iPSCs and differentiation to ROs and RPE

Dermal fibroblast lines were established from skin biopsies of 3 individuals with retinal dystrophy (one from IRDF-1 and two from IRDF-2). iPSCs were generated by reprogramming retinal dystrophy and control fibroblasts named BJ (ATCC CRL-2522), as described previously.[26] iPSCs were then differentiated to RPE following a previously described protocol.[27] iPSCs were also differentiated to 3D ROs as previously described, with some modifications.[28,29] Briefly, the cells were seeded on Geltrex-coated (Thermo Scientific) plates with mTESR Plus Medium (STEMCELL Technologies) until 90%–95% confluency. Essential 6 Medium (Thermo Scientific) was added to the culture for 2 days, followed by the addition of neural induction media (Advanced DMEM/F-12 [1:1], 1% N2 supplement, 2 mM GlutaMax, and 1% penicillin/streptomycin [Pen/Strep]). Cultures were treatment with 1.5 nM BMP4 (PeproTech) on day 6 of differentiation, with subsequent half-medium changes until day 16. Neuro retinal vesicles were excised and kept in 96-well plates for maturation. For retinal differentiation and maturation, a serum-free retinal differentiation medium was added (DMEM/F12 [3:1], 2% B27, 1% non-essential amino acids, and 1% Pen/Strep) for 6 days. The medium was then supplemented with 10% FBS, 100 μM taurine, and 2 mM GlutaMax. Retinoic acid (1 μM) was added on day 50. On day 70, N2 was added to the medium (RMM2), and the concentration of retinoic acid was reduced to 0.5 μM. To promote photoreceptor differentiation, the retinoic acid was removed from the medium on day 100.

RNA-seq

RNA sequencing (RNA-seq) was performed in fibroblasts, RPE, and ROs at day 150 of differentiation (day 150 ROs) from an affected male in each family and a male control individual. Total mRNA was isolated from the RNeasy Mini or Micro Kit (QIAGEN) using on-column DNase treatment (Promega) following the manufacturer's instructions. Samples were submitted in triplicate for RNA-seq to Otogenetics (USA). cDNA libraries were generated using random primed strand-specific synthesis (fibroblasts and RPE; TruSeq Stranded Total RNA Sample Prep Kit with Ribo-Zero H/M/R) or nondirectional poly(A) synthesis for low-input samples (day 150 ROs; Illumina, San Diego, CA, USA). Paired-end sequencing (100–125 bp) was performed on an HiSeq2500 Sequencer (Illumina), using the HiSeq SBS Kit v.4 (Illumina) with 30 million reads designated for fibroblasts and 100 million reads for RPE and ROs. Data quality assessment of RNA-seq reads was performed using FastQC (http://www.bioinformatics.babraham.ac.uk/projects/fastqc/). Residual Illumina adapters were

trimmed from raw RNA-seq reads using cutadapt (https://doi.org/10.14806/ej.17.1.200). STAR v.2.6.0c (https://doi.org/10.1093/bioinformatics/bts635) was subsequently used to align reads to a modified version of the human genome (hg38, Ensembl v.92) where an artificial chromosome was generated by inserting retinal dystrophy-specific SV sequences into the reference chrX. Alignment results were visually inspected as BAM files by using the IGV software (v.2.4) (http://software.broadinstitute.org/software/igv/).

Differential gene expression analysis

Fastq files containing bulk RNA-seq reads were aligned to a decoy-aware GENCODE v.43 hg38 reference index using Salmon v.1.10.1. Additional options "–validateMappings –gcBias –seqBias" were passed in for the Salmon alignment.[30,31]

Differential gene expression analysis of fibroblasts, iPSC-derived RPE, and ROs from the retinal dystrophy families and control groups were carried out using R v.4.2.1 and its associated packages (https://www.R-project.org/). Quantification data from Salmon were directly imported using tximeta v.1.16.1 into DESeq2 v.1.38.3 for differential gene expression analysis.[32,33] To check for batch effects, the data were first plotted on a principal-component analysis (PCA) graph after undergoing regularized logarithm (rlog) transformation. rlog-transformed quantification data were also used to produce a heatmap of Xq27.1 genes across different conditions and tissue types. Differential gene expression analysis on unnormalized Salmon pseudocounts was carried out by DESeq2, which assumes a negative binomial distribution for read counts and fits a generalized linear model for each gene.[32,33] Adjusted p values ($p$adj) were calculated using the independent hypothesis weighting (IHW) v.1.26.0 package to test the null hypothesis that differential gene expression is less than 1.5-fold between control and retinal dystrophy samples.[34] A $p$adj of <0.05 was used to filter for significant differentially expressed genes (DEGs). For both control to IRDF-1 and control to IRDF-2 comparisons, the control condition was used as the reference level. Shrunken log2 fold change (LFC) values were estimated using apeglm v.1.20.0.[35] These shrunken LFC values were used for plotting the DEGs based on the LFC.

RT-qPCR

To assess and validate the differential expression of genes implicated in the SVs, RT-qPCR was performed for non-retinal (fibroblasts) and retinal (RPE and day 150 ROs) tissues. Total RNA was extracted using the RNeasy Mini or Micro Kit (ROs) (QIAGEN) following the manufacturer's instructions. cDNA synthesis was performed using the Tetro cDNA Synthesis Kit (Bioline Reagents, London, UK). qPCR was completed using the SYBR Green method, carried out on a QuantStudio 6 Flex Real-Time PCR System (Applied Biosystems, Carlsbad, CA, USA) using LabTaq Green Hi Rox (Labtech, Heathfield, East Sussex, UK). Primers are listed in Tables S2 and S4. Relative gene expression levels were determined using the ΔΔCt method, compared to the reference genes *GAPDH* and/or *ACTIN*. GraphPad Prism v.8 (GraphPad Software) was used for statistical analyses and generating plots. Statistical analyses were performed using one-way ANOVA with Dunnett's correction.

Identification of *LINC00632* transcripts

To amplify linear *LINC00632* transcripts that could include the *CDR1as* exon and a promoter and start sites outside the *SOX3* TAD, control day 150 RO cDNA was amplified using primers designed to amplify transcripts from exon 1 of ENST00000602535.2 to the *CDR1as* exon. A forward primer spanning exon junction 1–3 was paired with a reverse primer in the *CDR1as* exon and the product nested using different reverse primers spanning alternate shorter versions of exon 3 (primers in Table S2). The products were gel purified and Sanger sequenced.

Preparation of Hi-C libraries

Hi-C-seq was performed on IRDF-1, IRDF-2, and control fibroblast lines. High-throughput chromosome conformation capture (Hi-C) libraries were processed as described previously.[36,37] Libraries were deep sequenced (~240 million fragments for fibroblasts and 320 million fragments for ROs) in a 100 bp paired-end run on a NovaSeq 6000 (Illumina). For each line, the Hi-C library was generated by pooling 4 technical replicates to ensure the high complexity of the sequencing library. Paired-end sequencing data were processed using the Juicer pipeline v.1.5.6, CPU version,[38] and Hi-C maps were created using a bin size with 10 kb resolution. Further information about the bioinformatics pipeline is described in Melo et al.[37]

CUT&Tag

Cleavage under targets and tagmentation sequencing (CUT&Tag) sequencing was performed on day 150 ROs from IRDF-2 and a control. One RO was used per sample. Samples were submitted in triplicate for CUT&Tag. Chromatin markers H3K27ac (active chromatin), H3K4Me1 (enhancers), and H3K4Me3 (active promoters) sequences were aligned to hg19 and lifted over to hg38. The chromatin marker signatures for regions of interest, including the Xq27.1 region and insertions, were visualized using the UCSC Genome Browser.

Identification of *miR-7* targets

hsa-miR-7-5p targets were selected using the miRTarBase website (https://awi.cuhk.edu.cn/~miRTarBase/miRTarBase_2025/php/index.php) and evidence in the literature.[39] A $p$adj of <0.05 was used to identify significantly DEGs. After a cross-reference with our RO RNA-seq expression data, 63 genes were identified. For both control to IRDF-1 and control to IRDF-2 comparisons, the control condition was used as the reference level. Shrunken LFC values were estimated using apeglm v.1.20.0.[35]

Results

X-linked retinal dystrophy families

We investigated two families, IRDF-1 (MEH-5421) and IRDF-2 (MAN-3539), with non-syndromic X-linked retinal degeneration and no molecular diagnosis. The pedigrees are shown in Figure 1. Affected members of the families were male with the exception of IV:13, who had an additional phenotype of variant Turner syndrome caused by a heterozygous deletion of chromosome Xp. Haplotype analysis showed the Xp deletion is in *trans* with the retinal dystrophy haplotype in this individual (data not shown). Ophthalmological examination of affected individuals in IRDF-1 revealed features of a slowly progressive cone, cone-rod, or macular dystrophy (Figures 1 and S1; Table S1). Eight affected subjects had foveal hypoplasia. OCT of the 2 younger subjects (IV:15 and IV:16) shows that the photoreceptor loss starts in the parafovea, and

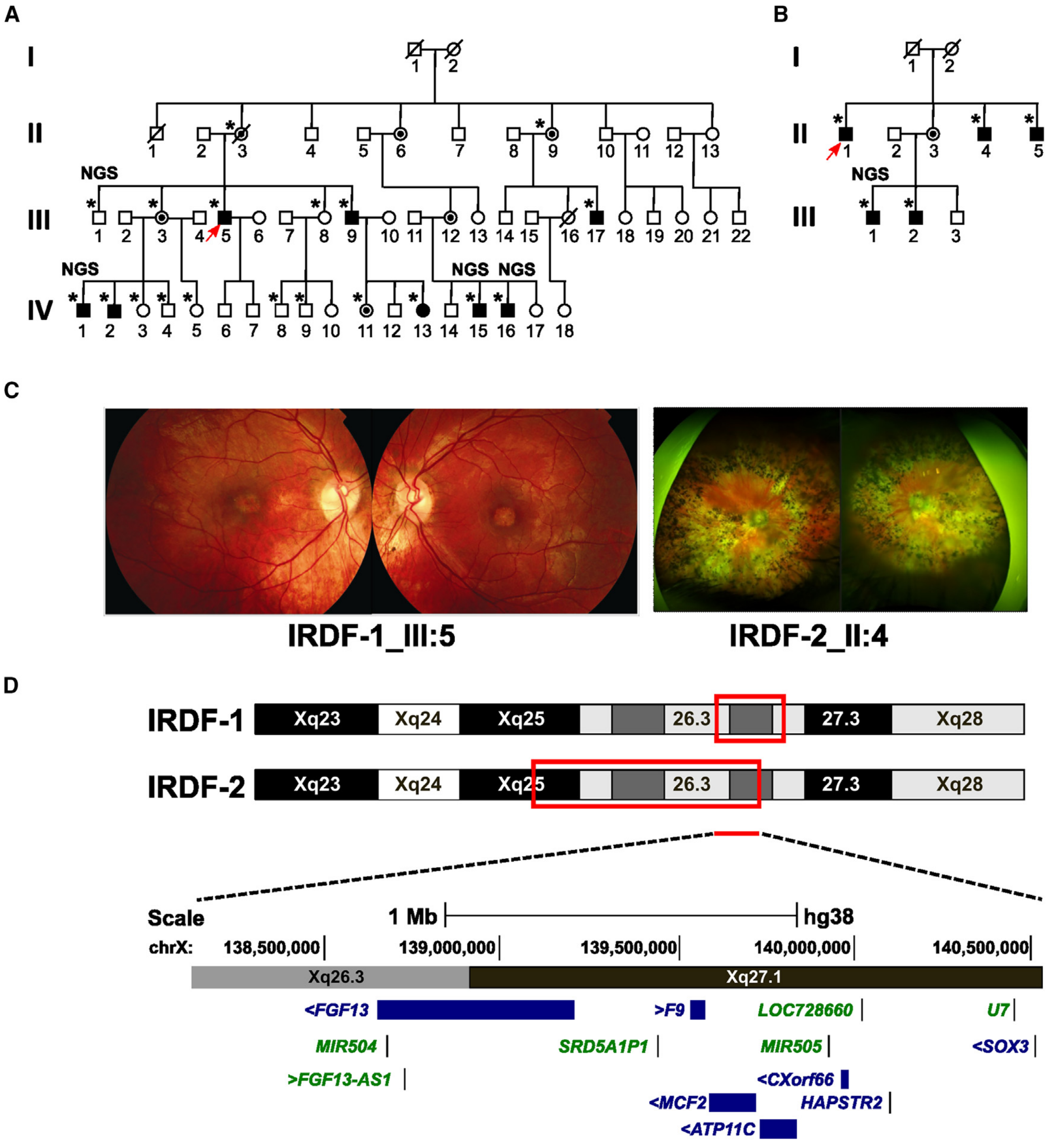

Figure 1. X-linked inherited retinal dystrophy in two unrelated families maps to Xq25–Xq27.2
(A) Pedigree structure of family IRDF-1.
(B) Pedigree of IRDF-2. Probands are indicated by an arrow. Individuals included in haplotype, segregation analysis, or NGS are indicated by an asterisk (*) and NGS, respectively.
(C) Retinal images of IRDF-1 III:5 (left with images of left and right eye) at age 39 years showing macular atrophy and IRDF-2 II:4 (right with images of left and right eye) at age 50 years showing peripheral retinal pigmentation and macular degeneration.
(D) Schematic showing overlapping loci for IRDF-1 and IRDF-2 on Xq (red boxes). The genes included in the overlap between the loci are shown on the bottom.

with fundus autofluorescence (FAF), the presence of hyper-autofluorescent rings and peripapillary atrophy was a common finding. Peri/paravascular atrophic changes were observed in 2 older subjects (III:5 and III:9). For the second family, IRDF-2, ophthalmological examination was suggestive of a rod-cone dystrophy, compatible with advanced retinitis pigmentosa with foveal involvement for II:2 (Figures 1 and S1; Table S1). Individual II:3 showed a milder phenotype with a tapetal-like reflex and peripheral degeneration.

Defining the X-linked retinal dystrophy locus

Following the exclusion of coding variants in X-linked genes known to cause retinal dystrophy (*RPGR* and *RP2*), the IRDF-1 locus was mapped by haplotype analysis to a 3.5 Mb region on Xq26.3–27.2 between markers DXS1062 and DXS1227 (Figures 1 and S2). The locus was subsequently refined to 1.6 Mb on Xq27.1–27.2 by SNV haplotyping from NGS data. WGS analysis of two affected individuals in IRDF-1 (IV:15 and IV:16) failed to identify any shared rare (MAF $<$ 0.001) candidate coding

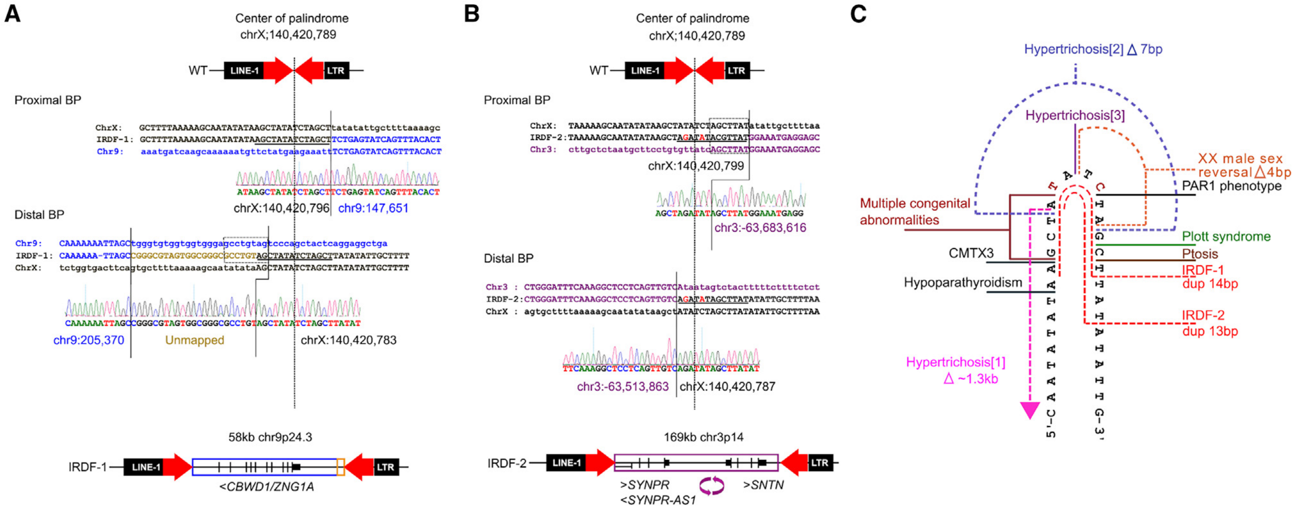

Figure 2. X-linked retinal dystrophy is associated with different inter-chromosomal insertions into a human-specific palindrome on Xq27.1

(A) Characterization of the proximal and distal breakpoints of the 9p24.3 insertion in IRDF-1. The Xq27.1 wild-type (WT) allele encompasses a 180 bp palindrome (red facing arrows) flanked by a long interspersed nuclear element (LINE-1) and long tandem repeat (LTR) (top). The center of the palindrome is shown with a dotted line. The 58 kb 9p24.3 insertion contains a partial sequence of *CBWD1/ZNG1A*. Reference sequences are shown above the chromatograms and color coded for chr9 (blue), unmapped (gold), and ChrX (black). Duplication of 14 bp of chrX is underlined. Regions of sequence homology are shown in a dotted box.

(B) Characterization of the proximal and distal breakpoints of the 3p14.2 insertion in IRDF-2. The WT and insertion alleles are depicted above and below the chromatograms. The 169 kb 3p14.2 insertion is inverted and inserted in the reverse orientation (semicircular arrows) and contains a partial sequence of *SYNPR*, *SYNPR-AS1*, and *SNTN*. Duplication of 13 bp of chrX is underlined. Regions of sequence homology are shown in a dotted box.

(C) Depiction of the human-specific palindrome on Xq27.1 showing the location of the retinal dystrophy insertions with respect to other inter-chromosomal insertions reported for different rare X-linked inherited conditions, including Charcot-Marie-Tooth disease type 3 (CMTX3). Image made in the same style as Figuera et al.[41] for consistency, incorporating additional X-linked disease-associated insertions including those reported here.

or non-coding SNVs within the locus. Comparative genomic hybridization with a dense X chromosome array excluded copy-number variants (CNVs) arising from the X chromosome (data not shown). For IRDF-2, the locus was mapped to a 13.6 Mb region on Xq25–Xq27.1 by SNP array genotyping (Figure S2). This locus partially overlapped the IRDF-1 locus (Figure 1). WES analysis did not identify any candidate rare coding or splice site variants within the mapped loci (MAF < 0.001).

Identification of inter-chromosomal insertions within a human-specific palindrome on Xq27.1

Next, we focused the analysis for IRDF-1 on a gap in the genome sequence within the locus. A poorly covered, camouflaged dark region on Xq27.1 (Figure S3) was found to correspond with a repeat-rich intergenic region containing a 180 bp human-specific palindrome. Long-range PCR was performed to bridge the gap using flanking PCR primers (supplemental methods; Table S2). Amplification was achieved in control and unaffected male samples but not affected males (Figure S3), suggesting disruption of the locus. DNA walking (supplemental methods; Table S2) across the locus from the telomeric end of the palindrome (affected individual IRDF-1 IV:1) revealed a distal breakpoint at chrX:140,420,783 (hg38) near the center of the palindrome. Here, the Xq27.1 sequence was linked to the 9p24.3 sequence via 23 bp of an intervening unmapped repeat sequence (Figure 2). This suggested the presence of an inter-chromosomal insertion of 9p24.3 into the palindrome. The proximal breakpoint of the insertion was identified by analysis of WGS data, which showed increased copy numbers of the first 10 exons of *CBWD1/ZNG1A* (MIM: 611078) on 9p24.3 (data not shown). Long-range PCR (primer pair chr9R1/HSPF; supplemental methods; Table S2) was then used to amplify the proximal breakpoint (chrX:140,420,796/chr9:147,651) (hg38). The breakpoints (chr9:147,651 and chr9:205,370) delineated an insertion of 58 kb of 9p24.3, which included exons 1–10 of *CBWD1/ZNG1A* (Figures 2 and S4). Breakpoint sequencing also revealed a 14 bp duplication of the center of the palindrome (chrX:140,420,783–140,420,796) with the duplicated sequence flanking the insertion (Figure 2). The unmapped breakpoint sequence showed homology (8 bp) to the 9p24.3 sequence and partial homology to the Xq27.1 sequence at the distal breakpoint, suggesting an underlying microhomology-mediated break-induced replication (MMBIR) mechanism for this SV (Figure 2).[40]

Since the IRDF-2 locus also spans the palindrome (Figure 1), we questioned whether a similar genomic mechanism could be the cause of retinal dystrophy in this family. PCR amplification across the palindrome in affected individuals could not be achieved. DNA walking from the Xq27.1 telomeric and centromeric flanking regions of the palindrome revealed a distal breakpoint

at chrX:140,420,787 joined to chromosome 3p14.2 (chr3:-63,513,863, hg38) and a proximal breakpoint at chrX:140,420,799/chr3:-63,683,616 (Figure 2). The insertion in IRDF-2 is an ~169 kb inverted insertion of 3p14.2 flanked by a duplication of 13bp from chrX:140,420,787–140,420,799 near the center of the palindrome. At the proximal breakpoint, the chrX sequence shows 7 bp homology to the 3p14.2 insertion sequence, indicating a similar underlying MMBIR mechanism. This insertion contains three terminal exons of *SYNPR*, the 5′ exon and upstream region of *SYNPR-AS1*, and a complete copy of *SNTN* (MIM: 617832) (Figures 2 and S4).

The possibility that the insertions were benign and in linkage disequilibrium (LD) with a causative variant was considered, but no rare candidate variants were identified. Importantly, the human-specific palindrome on Xq27.1 is a recognized mutation hotspot that has previously been associated with rare X-linked phenotypes and unique inter-chromosomal insertions within the palindrome, including congenital generalized hypertrichosis (CGH; HTC2 [MIM: 307150]), Charcot-Marie-Tooth neuropathy (CMTX3 [MIM: 302802]), congenital ptosis (PTOSX [MIM: 300245]), hypoparathyroidism (HYPX [MIM: 307700]), sex reversal (SRXX3 [MIM: 300833]), an isolated bilateral vocal cord paralysis also known as Plott syndrome (MIM: 308850), and two different multisystem congenital disorders.[41–50] The precise position of the two retinal dystrophy insertions that we identified within the palindrome, compared to the inter-chromosomal insertions associated with other rare X-linked conditions, is shown in Figure 2. We therefore hypothesized that the two different retinal dystrophy insertions were causative, with a convergent mechanism leading to the dysregulation of genes within, or flanking, the insertion.

Transcriptome analysis of cell models derived from affected individuals

The inter-chromosomal insertions could lead to dysregulated gene expression by (1) disrupting normal gene expression from dosage-sensitive genes within the insertions, (2) a position effect, (3) alternative splicing, (4) disruption of 3D chromatin architecture, or (5) the introduction of regulatory elements that interact with nearby genes in a tissue-specific manner. To explore these hypotheses, we reprogrammed fibroblasts from affected individuals in both families into iPSCs. Subsequently, we differentiated these iPSCs to 3D ROs and RPE (Figure S5). RNA-seq was generated for fibroblasts, day 150 ROs, and RPE (supplemental methods). RNA-seq profiles of marker gene expression, including *RHO* (MIM: 180380), *NR2E3* (rods) (MIM: 604485), *ARR3* (MIM: 301770) and *OPN1SW* (cones) (MIM: 613522), *TYR* (MIM: 606933), and *LRAT* (RPE) (MIM: 604863), further confirmed that both tissue types were differentiated successfully (Figure S6).

In order to assess any aberrant expression of genes within the insertions, in addition to Xq27.1 genes flanking the palindrome, we generated artificial reference X chromosomes containing the insertions. There was no significant differential expression of 9p24.3- or 3p14.2-derived insertion transcripts in IRDF-1- or IRDF-2-derived fibroblasts, RPE, or ROs, respectively. The only exception was downregulation of *SYNPR* in IRDF-2 RPE, the 3′ end of which is included in the 3p14.2 insertion (Figures 3 and S4; Table S3). The expression of *SYNPR* was, however, very low in RPE (<10 transcripts per million [TPM] in controls: Table S3); therefore, we concluded that dysregulation of genes within the insertions is unlikely to be the mechanism of disease.

Next, we analyzed Xq27.1 coding transcripts flanking the insertions, including *FGF13* (MIM: 300070), *MCF2* (MIM: 311030), *ATP11C* (MIM: 300516), *SOX3* (MIM: 313430), *CDR1* (MIM: 302650), *SPANXB1* (MIM: 300669), and *LDOC1* (MIM: 300402) (Tables 1 and S3; Figure 3). We found no evidence of differential expression in RPE or ROs derived from affected individuals compared to control individuals, with the exception of *SOX3*, which was significantly downregulated in IRDF-1 ROs but not in IRDF-2. The level of *SOX3*, however, was extremely low in day 150 ROs (mean of 3.14 TPM in control ROs) and below the limit of detection by RT-qPCR. The low level of expression combined with the absence of any retinal dystrophy associated with *SOX3* deficiency suggested it was also unlikely to be the cause of IRD.[51–55] *FGF13* was upregulated in IRDF-1 fibroblasts; however, there was no significant difference in expression in RPE or ROs.

A striking finding was the high level of expression of long non-coding RNA (lncRNA) *LINC00632* in control ROs, suggesting that *LINC00632* has an important, as-yet undefined, role in the retina. Comparative analysis of day 150 RO RNA-seq data showed that *LINC00632* was significantly upregulated in IRDF-1 and IRDF-2 ROs compared to controls (Table 1; Figure 3). Conversely, it was significantly downregulated in RPE from affected individuals in both families compared to control individuals. Based on these findings, we reasoned that tissue-specific dysregulation of *LINC00632* could represent a potential convergent mechanism for retinal dystrophy, prompting further experimental investigations to explore this hypothesis.

3D chromatin architecture of the Xq27.1 retinal dystrophy locus

To investigate the 3D conformation of the Xq27.1 retinal dystrophy locus, we interrogated publicly available Hi-C data of the region and performed Hi-C in control human fibroblasts and day 150 ROs (supplemental methods; Figure 3). No major differences in 3D chromatin structure were identified, indicating the TADs are largely conserved between tissues. The Xq27.1 insertion locus is situated 80 kb downstream of *SOX3*, an embryonic transcription factor gene that has a well-characterized *cis*-regulatory region. We observed that *SOX3* lies within a TAD that harbors two protein-coding genes (*HAPSTR2* and *SOX3*), two non-coding RNA genes (lncRNA *LOC728660* and *U7*), and the

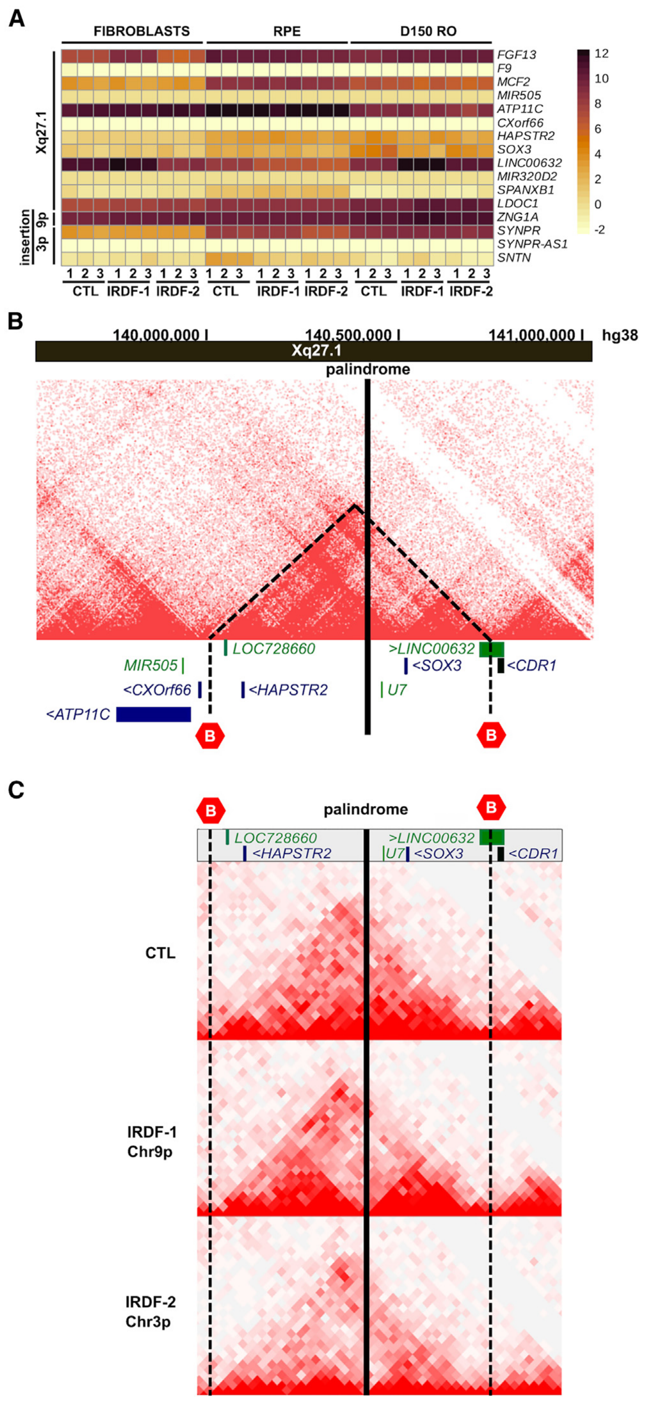

Figure 3. Regional gene expression profiles and 3D chromatin architecture

(A) Heatmap of gene expression profiles generated from RNA-seq data. Genes on Xq27.1 flanking the insertions and within the insertions in IRDF-1 (9p) and IRDF-2 (3p) are shown to the right of the heatmap. Differential expression was assessed in fibroblasts, RPE, and day 150 ROs (in triplicate) derived from control (CTL) and affected individuals.

(B) CTL day 150 RO Hi-C showing the location of the palindrome within a TAD (triangle with dotted lines) encompassing *SOX3* and *LINC00632* and position of the CTCF-associated TAD boundaries. *LINC00632* spans the distal TAD boundary.

(C) CTL fibroblast Hi-C showing the TAD and boundaries are conserved across tissues (top). The *SOX3* TAD boundaries appear undisrupted by the insertions in IRDF-1 and IRDF-2.

5′ region of *LINC00632*. The *SOX3* TAD is separated from neighboring TADs by CTCF-associated TAD boundaries in the classical convergent orientation (Figures 3 and S7). As *LINC00632* lies largely outside the *SOX3* TAD, we hypothesized that the dysregulation of *LINC00632* resulted either (1) from the interaction of retinal enhancers within the insertions on *LINC*00632 promoters inside the TAD or (2) through disruption of the telomeric *SOX3* TAD boundary, enabling the interaction of insertions enhancers with promoters normally situated outside the TAD.

To explore these hypotheses, we performed Hi-C on IRDF-1 and IRDF-2 fibroblasts for comparison to control fibroblasts. Here, we observed the *SOX3* proximal and distal TAD boundaries remained intact (Figure 3), suggesting that the most likely mechanism driving the upregulation of linear *LINC00632* expression in ROs is the interaction of retina-specific enhancers within the insertions with the *LINC00632* promoter inside the TAD.

Retinal dystrophy-associated inter-chromosomal insertions contain retina-specific enhancers

Interrogation of ENCODE for functional regulatory elements within the IRDF-1 and IRDF-2 insertions (9p24.3 and 3p14.2) showed that they each include *cis*-regulatory enhancer elements. These ENCODE enhancers corresponded to photoreceptor-specific *OTX2* and *CRX* transcription factor signatures in retina (Figure S8),[24] supporting our hypothesis that retinal enhancers within the insertions could be driving the dysregulation of *LINC00632*.

To further investigate the retinal chromatin architecture at the *LINC00632* locus, we performed CUT&Tag in day 150 control ROs to assess the activating histone modifications H3K27ac, H3K4me1, and H3Kme3. H3K27ac (active chromatin/enhancers) and H3K4me1 (poised enhancers) peaks were found within and outside of the *SOX3* TAD boundary and corresponded to different *LINC00632* transcripts. H3K4me3 peaks were also enriched at transcription start sites and were spread more broadly (Figure 4).

Tissue-specific dysregulation of *LINC00632* and *CDR1as/ciRS-7* as a convergent mechanism for Xq27.1 retinal dystrophy

LINC00632 has generated recent interest because of its association with a downstream circular RNA (circRNA) *CDR1as* (cerebellar degeneration-related protein 1 antisense transcript [MIM: 300898]), also known as the circular RNA sponge for *miR-7* (*ciRS-7*).[56] *CDR1as/ciRS-7* is transcribed in antisense orientation to the putative protein-coding gene *CDR1* and forms a covalently closed single-stranded circRNA. *LINC00632* and *CDR1as/ciRS-7*, both on the plus DNA strand of Xq27.1, were previously thought to be adjacent genes. It is now clear that *CDR1as/ciRS-7* shares promoters with *LINC00632* and is alternatively spliced from linear *LINC00632* pre-RNA to form a circular isoform *CDR1as/ciRS-7* (Figure 4).[56] *CDR1as/ciRS-7* is formed when the downstream 5′ splice donor of the exon is joined by "back-splicing" to an upstream 3′ splice acceptor, leaving

Table 1. **Differential expression of genes on Xq27.1 flanking the inter-chromosomal insertions in three different tissues**

Genes	Fibroblasts				RPE				Day 150 retinal organoids			
	IRDF-1		IRDF-2		IRDF-1		IRDF-2		IRDF-1		IRDF-2	
	L2FC	*padj*	L2FC	*padj*	L2FC	*padj*	L2FC	*padj*	L2FC	*padj*	L2FC	*padj*
FGF13	3.04[a]	3.87E−24[a]	−1.48	0.48	−0.06	1	−0.62	1	0.83	1	1.21	0.05
SRD5A1P1	N/D	N/D	N/D	N/D	N/D	N/D	N/D	N/D	N/D	N/D	N/D	N/D
F9	−1.38	1	−0.36	1	N/D	N/D	N/D	N/D	1.85	1	N/D	N/D
MCF2	−0.33	1	−0.31	1	0.41	1	−0.13	1	−0.83	1	−0.92	0.70
MIR505	N/D	N/D	N/D	N/D	N/D	N/D	N/D	N/D	N/D	N/D	N/D	N/D
ATP11C	0.05	1	0.68	1	−0.34	1	−0.32	1	−0.28	1	−1.10	1
CXorf66	N/D	N/D	N/D	N/D	N/D	N/D	N/D	N/D	2.63	1	N/D	N/D
LOC728660	N/D	N/D	N/D	N/D	N/D	N/D	N/D	N/D	N/D	N/D	N/D	N/D
HAPSTR2	N/D	N/D	N/D	N/D	1.01	1	0.36	1	−0.93	0.93	−0.13	0.61
SOX3	0.21	1	1.26	1	−0.26	1	−1.18	1	−3.11[a]	0.00[a]	−1.820	0.08
LINC00632	0.632	1	−2.67[a]	1.14E−08[a]	−2.11[a]	1.97E−09[a]	−3.04[a]	1.41E−15[a]	2.99[a]	1.50E−16[a]	1.35[a]	0.02[a]
MIR320D2	N/D	N/D	N/D	N/D	N/D	N/D	N/D	N/D	N/D	N/D	N/D	N/D
SPANXB1	−0.74	1	0.28	1	0.37	1	0.79	1	N/D	N/D	0.10	1
LDOC1	0.82	1	1.34	0.06	0.11	1	0.18	1	0.77	1	0.94	0.09

L2FC, log2 fold change based on maximum likelihood estimation (MLE); *p*adj, adjusted *p* value; N/D, not detected.
[a]Significantly upregulated and downregulated values (*p*adj $<$ 0.05).

no exposed ends. The covalent 3′-5′ phosphodiester bond of the back-splice junction results in a high level of circRNA stability compared to linear RNA transcripts. *CDR1as/ciRS-7* is highly conserved in mammals, with high abundance in excitatory neurons and not detected as a linear transcript.[57] We identified RNA-seq reads in our ROs aligned to the *CDR1as/ciRS-7* locus that were spliced to upstream *LINC00632* exons, while other transcripts had several base pairs of an unaligned sequence at the 5′ or 3′ ends of the *CDR1as/ciRS-7* transcripts that were identified as back-splice junctions (Figure 4).

Given the observation that *LINC00632* is dysregulated (upregulated) in RNA-seq data from IRDF-1 and IRDF-2 ROs compared to controls, the upregulation of linear *LINC00632* transcripts that do not include the *CDR1as/ciRS-7* exon (NR_028344.1 and GENCODE transcripts ENST00000649192.2, ENST00000659304.1, and ENST00000649335.2) (hg38) was confirmed by RT-qPCR using transcript-specific primers (Figure 4; Table S4). Next, we investigated the differential expression of *CDR1as/ciRS-7* in fibroblasts, day 150 ROs, and RPE by back-splice junction RT-qPCR. In fibroblasts, *CDR1as/ciRS-7* was significantly upregulated in IRDF-1 and IRDF-2 compared to control individuals. In contrast, in RPE and ROs, *CDR1as/ciRS-7* was downregulated, suggesting a convergent, tissue-specific dysregulation in the retinal dystrophy models.

CDR1as/ciRS-7 expression in retina is driven by two promoters on different sides of the *SOX3* TAD boundary

An inverse relationship between linear *LINC00632* and *CDR1as/ciRS-7* was identified in the *Cdr1as* knockout (KO) mouse, raising the possibility that *CDR1as/ciRS-7* directly regulates its host transcript through sequestering or competing for splicing enhancers.[56,57] However, if *CDR1as/ciRS-7* transcription originates solely from the *LINC00632* promoter within the *SOX3* TAD, then we would expect it to be upregulated in our retinal dystrophy RO models, owing to the upregulation of the parental linear isoforms. We therefore investigated if a different mechanism controls the level of *CDR1as/ciRS-7*.

The *CDR1as/ciRS-7* exon lies outside the TAD boundaries; however, two promoters drive the expression of parental linear *LINC00632*.[56] One of these *LINC00632* promoters (P1) lies within the *SOX3* TAD and has been shown by chromatin immunoprecipitation (ChIP) and RNA-seq studies in mouse brain to promote the expression of *LINC00632*. The other promoter (P2) lies at the 5′ end of a short linear transcript (GENCODE: ENST00000602535.2) outside the TAD (Figure 4). CUT&Tag confirmed the presence of two active promoters in control ROs (Figure 4). Using cDNA derived from control day 150 ROs, we identified two short transcripts containing the *CDR1as/ciRS-7* exon: *NR_190227.1* and a new transcript with a larger exon 2 (Figures 4 and S9). These retinal isoforms provide additional evidence for shorter linear *LINC00632* parental transcripts of *CDR1as/ciRS-7* associated with a second promoter outside the *SOX3* TAD boundary. These promoters, separated by the *SOX3* TAD boundary, could therefore be differentially regulated by specific enhancers or the chromatin state of their promoters. While retinal enhancers within the insertions described here are likely only to affect transcripts driven by the centromeric promoter (P1), Hi-C data from human

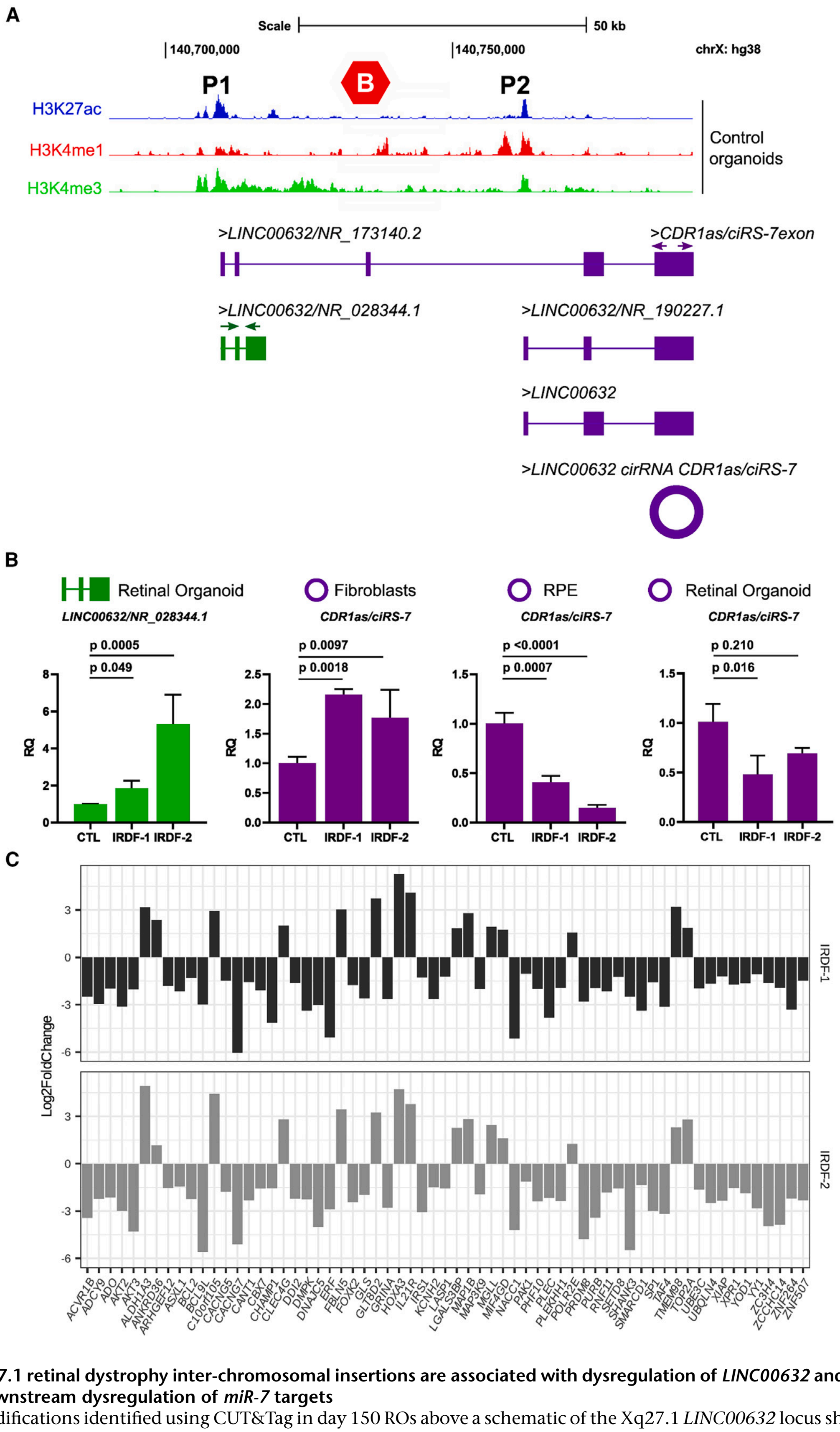

Figure 4. Xq27.1 retinal dystrophy inter-chromosomal insertions are associated with dysregulation of *LINC00632* and *CDR1as/ciRS-7* resulting in downstream dysregulation of *miR-7* targets

(A) Histone modifications identified using CUT&Tag in day 150 ROs above a schematic of the Xq27.1 *LINC00632* locus showing location of transcripts with respect to the *SOX3* distal TAD boundary (CTCF, red hexagon) and the two promoters, P1 and P2, on different sides of the TAD boundary.

(B) RT-qPCR of linear (green) and circular (purple) *LINC00632* expression. Primer positions are shown by green and purple arrows on the corresponding transcripts. Linear isoform *LINC00632/NR_028344.1* is significantly upregulated in both IRDF-1 and IRDF-2 day 150 ROs

(legend continued on next page)

retina tissue show a loop between the promoters of the long and short *CDR1as/ciRS-7*-containing isoforms indicating they could regulate one another.[24,25]

Downstream consequence of dysregulated *LINC00632* and *CDR1as/ciRS-7*

Cytoplasmic *CDR1as/ciRS-7* functions as a gene regulator through the binding of microRNAs (miRNAs) and RNA-binding proteins, such as Argonaute proteins and IGF2BP3.[57,58] *CDR1as/ciRS-7* is highly conserved in mammals and abundant in brain, particularly in the excitatory neurons, where it regulates neuronal activity and synaptic transmission.[57,59–61] In neural tissues, *CDR1as/ciRS-7* acts as a *miR-7* reservoir, with over 70 binding sites for *miR-7*, thus preventing the interaction and suppression of *miR-7* target transcripts.[60,62,63]

To explore the consequence of downregulation of *CDR1as/ciRS-7* in IRDF-1 and IRDF-2, we investigated the relative expression of previously validated downstream targets of *miR-7* (in retina and brain) in both IRDF-1 and IRDF-2 ROs compared to controls. Strikingly, we found that these target transcripts were significantly dysregulated in IRDF-1 and IRDF-2 ROs compared to controls (Figure 4; Table S5). The majority of the downstream target genes were downregulated, and the direction of dysregulation was consistent in both IRDF-1 and IRDF-2 ROs (Figure 4; Table S5), supporting the downregulation of *CDR1as/ciRS-7* and increased activity of *miR-7* as potential mechanisms of disease.

Discussion

This study investigated the cause of X-linked (XL) retinal dystrophy in two families without a molecular diagnosis but with overlapping mapped loci on Xq26–27.2. This region on Xq encompasses two previous reported loci in single families with IRDs, CORDX2 (MIM: 300085) and RP24 (MIM: 300155), for which the causative variants have not been identified. We speculate that the cause of disease in these families could be different inter-chromosomal insertions within the human-specific palindrome on Xq27.1, as described here.

The palindrome on Xq27.1 is a recognized mutation hotspot that has previously been associated with unique inter-chromosomal insertions within the palindrome, associated with rare X-linked phenotypes. X-linked congenital generalized hypertrichosis (CGH) has been reported in three unrelated pedigrees with different inter-chromosomal insertions, while other X-linked-associated phenotypes include congenital hypoparathyroidism (HPT), ptosis (PTOSX), a disorder of sexual development (DSD), hereditary sensory motor neuropathy (CMTX3), and a congenital multisystem skeletal disorder (Figure 2).[41–47,50,64] The underlying mechanism of disease for these conditions has yet to be determined, although the differential expression of nearby genes has been implicated in some studies: *FGF13* in XL-CGH and *SOX3* in XL-HPT and XL-DSD.[43,47,64] The precise mechanism whereby the respective insertions affect the regulation of these genes has not been explored.

SOX3 encodes a member of the SOX (SRY-related HMG-box) family of transcription factors and is involved in the regulation of embryonic development and the determination of cell fate. Owing to its proximity to the insertions and the palindrome, its extensive *cis*-regulatory region, and its inclusion within the TAD, *SOX3* was considered a candidate in our families. However, our retinal dystrophy families showed no congenital abnormality and no clinical features consistent with *SOX3* dysregulation.[51–55] Transcriptomics showed extremely low expression of *SOX3* in our control and retinal dystrophy ROs, with small but significant differences in expression only in IRDF-1. Hi-C data confirmed the boundaries of the *SOX3* TAD were undisturbed by the insertions. CUT&Tag of ROs also showed no clear evidence of altered 3D chromatin architecture in the region, suggesting that regulation of *SOX3* was largely unaltered. The expression of other genes in the region, including *FGF13*, was also not significantly affected by the insertions.

LINC00632 is the only gene within the *SOX3* TAD that shows high expression in neural tissue. The high level of expression of linear *LINC00632* was evident in our ROs with the expression of multiple isoforms, including those containing the *CDR1as/ciRS-7* exon. Although *CDR1as/ciRS-7* lies outside the TAD boundaries, an upstream promoter shared with linear *LINC00632* and associated with expression of the circular *CDR1as/ciRS-7* is within the TAD and drives the expression of *LINC00632* through the boundary. Hi-C data show that the promoter is present within the same TAD as the insertions and would, therefore, be available for interactions with ectopic retinal enhancers. Importantly, our RNA-seq and RT-qPCR data analysis revealed tissue-specific dysregulation of *LINC00632* and *CDR1as/ciRS-7*, suggesting this is the most likely cause of retinal degeneration.

The upregulation of linear *LINC00632* transcripts in IRDF-1 and IRDF-2 driven by the promoter within the TAD suggests this is due to ectopic interaction with retinal enhancers within the insertions. We were able to confirm significant upregulation of short linear transcripts, which do not include the *CDR1as/ciRS-7* exon. In addition, we identified a new transcript containing this exon that is

compared to controls. In contrast, circular *CDR1as/ciRS-7* is downregulated in ROs and RPE and upregulated in fibroblasts for both IRDF-1 and IRDF-2, demonstrating the tissue specificity of this dysregulation. Error bars are ± SD; p values were calculated using one-way ANOVA with Dunnett's correction.
(C) Interrogation of RO RNA-seq data for validated *miR-7* targets (shown on the x axis) revealed a strikingly consistent pattern of significant up- and downregulation across both families (Table S5).

expressed in ROs. There is some evidence to suggest that *CDR1as/ciRS-7* has a role in regulating linear *LINC00632* transcripts.[56,57,65] However, our data suggest that the downregulation of *CDR1as/ciRS-7* is likely to be secondary to the upregulation of linear *LINC00632*, as a result of either enhancer-specific upregulation of transcripts that reduce circRNA splicing or differential expression of isoforms containing the *CDR1as/ciRS-7* exon controlled by different promoters dependent on the 3D chromatin state. The identification of a new short linear transcript in ROs that contains the *CDR1as/ciRS-7* exon with an active promoter outside of the *SOX3* TAD suggests that it is differentially regulated and may therefore have a different function from isoforms driven by the centromeric *LINC00632* promoter. The downstream consequence of reduced levels of *CDR1as/ciRS-7* would be predicted to be increased *miR-7* availability and the downregulation of *miR-7* targets, and the majority of *miR-7* target transcripts were decreased, but some targets were increased. The reason is unclear and requires further exploration; however, in some contexts, miRNAs can also upregulate their targets. Neuronal miRNAs, in particular *miR-9* and *miR-124*, can exhibit diverse context-dependent functions.[66] *miR-7* may also act in a context-dependent manner via interaction with *LINC00632* and its circular isoform acting as either a competing endogenous RNA or an *miR-7* stabilizer.[57]

Although we have identified a potential convergent and tissue-specific mechanism of disease for retinal dystrophy-associated inter-chromosomal insertions, it is not clear if this is driven by the upregulation of linear *LINC00632*, the downregulation of *CDR1as/ciRS-7*, or both, as they may act synergistically.

Our study highlights the importance of complex SVs in human disease and the use of appropriate models to study their consequences and expands the phenotypic association of inter-chromosomal insertions within the palindrome at Xq27.1. A limitation of short-read NGS is that this repetitive region appears as a gap and is not aligned, so it is likely that other rare X-linked conditions could be attributable to inter-chromosomal insertions within the palindrome.

Transcripts derived from the *LINC00632* locus have an as-yet undefined role in the retina but are highly expressed. The complex alternative splicing of the *LINC00632* locus into linear and circular transcripts warrants further investigation. Our work offers insights into the consequences of insertions that affect 3D chromatin architecture by creating the opportunity for ectopic contacts between enhancers and genes without affecting the TAD structure. It also provides a template for the discovery of the pathomechanisms underlying other inter-chromosomal insertion phenotypes within the palindrome at this Xq27.1 locus.

Data and code availability

NGS data supporting the current study have not been deposited in a public repository because of consent and ethical considerations. Although not publicly available, all data are available upon reasonable request to the corresponding authors.

Acknowledgments

This research was supported by the National Institute for Health Research Biomedical Research Centre at Moorfields Eye Hospital and UCL Institute of Ophthalmology (A.J.H., J.C.G., M.E.C., M.M., N.C., C.G.), the National Institute for Health and Care Research (NIHR) Manchester Biomedical Research Centre (BRC) (NIHR203308), the Medical Research Council UK (A.J.H., M.E.C.), the Wellcome Trust (M.E.C.), Retina UK and Fight for Sight UK IRDC (A.J.H., J.C.G., G.C.B., R.L.T., M.E.C., M.M.), and Moorfields Eye Charity (M.E.C.). We thank the families for their participation in this research and Beverley Scott for DNA extraction.

Author contributions

Conceptualization, J.C.G., A.J.H., and M.E.C.; experimentation and data generation, J.C.G., K.J., D.O., U.S.M., J.J., R.G., K.Z., K.-L.H., A.L., R.L.T., and O.F.; interpretation and analysis of data, J.C.G., A.J.H., D.O., U.S.M., J.J., N.C., C.G., M.P., S.M., and M.E.C.; clinical resources and clinical data, M.M., G.C.B., A.T.M., and M.G.; writing – original draft, J.C.G., A.J.H., and M.E.C. All authors reviewed and edited the manuscript.

Declaration of interests

The authors declare no competing interests.

Web resources

FastQC, http://www.bioinformatics.babraham.ac.uk/projects/fastqc/
GENCODE, https://www.gencodegenes.org
IGV, http://software.broadinstitute.org/software/igv/
miRTarBase, https://awi.cuhk.edu.cn/~miRTarBase/miRTarBase_2025/php/index.php
OMIM, https://www.omim.org
RegRet, http://genome.ucsc.edu/s/stvdsomp/RegRet
UCSC, https://genome.ucsc.edu/

Supplemental information

Supplemental information can be found online at https://doi.org/10.1016/j.ajhg.2025.01.007.

Received: October 15, 2024
Accepted: January 6, 2025
Published: January 31, 2025

References

1. Di Resta, C., Galbiati, S., Carrera, P., and Ferrari, M. (2018). Next-generation sequencing approach for the diagnosis of human diseases: open challenges and new opportunities. EJIFCC *29*, 4–14.
2. Ellingford, J.M., Barton, S., Bhaskar, S., O'Sullivan, J., Williams, S.G., Lamb, J.A., Panda, B., Sergouniotis, P.I., Gillespie, R.L., Daiger, S.P., et al. (2016). Molecular findings from 537

individuals with inherited retinal disease. J. Med. Genet. *53*, 761–767. https://doi.org/10.1136/JMEDGENET-2016-103837.

3. Haer-Wigman, L., van Zelst-Stams, W.A., Pfundt, R., Van Den Born, L.I., Klaver, C.C., Verheij, J.B., Hoyng, C.B., Breuning, M.H., Boon, C.J., Kievit, A.J., et al. (2017). Diagnostic exome sequencing in 266 Dutch patients with visual impairment. Eur. J. Hum. Genet. *25*, 591–599. https://doi.org/10.1038/ejhg.2017.9.
4. Jespersgaard, C., Fang, M., Bertelsen, M., Dang, X., Jensen, H., Chen, Y., Bech, N., Dai, L., Rosenberg, T., Zhang, J., et al. (2019). Molecular genetic analysis using targeted NGS analysis of 677 individuals with retinal dystrophy. Sci. Rep. *9*, 1219. https://doi.org/10.1038/S41598-018-38007-2.
5. Verbakel, S.K., van Huet, R.A.C., Boon, C.J.F., den Hollander, A.I., Collin, R.W.J., Klaver, C.C.W., Hoyng, C.B., Roepman, R., and Klevering, B.J. (2018). Non-syndromic retinitis pigmentosa. Prog. Retin. Eye Res. *66*, 157–186. https://doi.org/10.1016/J.PRETEYERES.2018.03.005.
6. Zenteno, J.C., García-Montaño, L.A., Cruz-Aguilar, M., Ronquillo, J., Rodas-Serrano, A., Aguilar-Castul, L., Matsui, R., Vencedor-Meraz, C.I., Arce-González, R., Graue-Wiechers, F., et al. (2020). Extensive genic and allelic heterogeneity underlying inherited retinal dystrophies in Mexican patients molecularly analyzed by next-generation sequencing. Mol Genet Genomic Med *8*, e1044. https://doi.org/10.1002/MGG3.1044.
7. Chaisson, M.J.P., Sanders, A.D., Zhao, X., Malhotra, A., Porubsky, D., Rausch, T., Gardner, E.J., Rodriguez, O.L., Guo, L., Collins, R.L., et al. (2019). Multi-platform discovery of haplotype-resolved structural variation in human genomes. Nat. Commun. *10*, 1784. https://doi.org/10.1038/S41467-018-08148-Z.
8. Sudmant, P.H., Rausch, T., Gardner, E.J., Handsaker, R.E., Abyzov, A., Huddleston, J., Zhang, Y., Ye, K., Jun, G., Fritz, M.H.Y., et al. (2015). An integrated map of structural variation in 2,504 human genomes. Nature *526*, 75–81. https://doi.org/10.1038/NATURE15394.
9. Collins, R.L., Brand, H., Karczewski, K.J., Zhao, X., Alföldi, J., Francioli, L.C., Khera, A.V., Lowther, C., Gauthier, L.D., Wang, H., et al. (2020). A structural variation reference for medical and population genetics. Nature *581*, 444–451. https://doi.org/10.1038/S41586-020-2287-8.
10. Pauper, M., Kucuk, E., Wenger, A.M., Chakraborty, S., Baybayan, P., Kwint, M., van der Sanden, B., Nelen, M.R., Derks, R., Brunner, H.G., et al. (2021). Long-read trio sequencing of individuals with unsolved intellectual disability. Eur. J. Hum. Genet. *29*, 637–648. https://doi.org/10.1038/s41431-020-00770-0.
11. Brandler, W.M., Antaki, D., Gujral, M., Kleiber, M.L., Whitney, J., Maile, M.S., Hong, O., Chapman, T.R., Tan, S., Tandon, P., et al. (2018). Paternally inherited cis-regulatory structural variants are associated with autism. Science *360*, 327–331. https://doi.org/10.1126/SCIENCE.AAN2261.
12. Han, L., Zhao, X., Benton, M.L., Perumal, T., Collins, R.L., Hoffman, G.E., Johnson, J.S., Sloofman, L., Wang, H.Z., Stone, M.R., et al. (2020). Functional annotation of rare structural variation in the human brain. Nat. Commun. *11*, 2990. https://doi.org/10.1038/s41467-020-16736-1.
13. Redin, C., Brand, H., Collins, R.L., Kammin, T., Mitchell, E., Hodge, J.C., Hanscom, C., Pillalamarri, V., Seabra, C.M., Abbott, M.A., et al. (2017). The genomic landscape of balanced cytogenetic abnormalities associated with human congenital anomalies. Nat. Genet. *49*, 36–45. https://doi.org/10.1038/NG.3720.
14. Ebbert, M.T.W., Jensen, T.D., Jansen-West, K., Sens, J.P., Reddy, J.S., Ridge, P.G., Kauwe, J.S.K., Belzil, V., Pregent, L., Carrasquillo, M.M., et al. (2019). Systematic analysis of dark and camouflaged genes reveals disease-relevant genes hiding in plain sight. Genome Biol. *20*, 97. https://doi.org/10.1186/S13059-019-1707-2.
15. Stankiewicz, P., and Lupski, J.R. (2010). Structural Variation in the Human Genome and its Role in Disease. Annu. Rev. Med. *61*, 437–455. https://doi.org/10.1146/annurev-med-100708-204735.
16. Lupiáñez, D.G., Kraft, K., Heinrich, V., Krawitz, P., Brancati, F., Klopocki, E., Horn, D., Kayserili, H., Opitz, J.M., Laxova, R., et al. (2015). Disruptions of Topological Chromatin Domains Cause Pathogenic Rewiring of Gene-Enhancer Interactions. Cell *161*, 1012–1025. https://doi.org/10.1016/j.cell.2015.04.004.
17. Spielmann, M., Lupiáñez, D.G., and Mundlos, S. (2018). Structural variation in the 3D genome. Nat. Rev. Genet. *19*, 453–467. https://doi.org/10.1038/s41576-018-0007-0.
18. Turner, T.N., Hormozdiari, F., Duyzend, M.H., McClymont, S.A., Hook, P.W., Iossifov, I., Raja, A., Baker, C., Hoekzema, K., Stessman, H.A., et al. (2016). Genome Sequencing of Autism-Affected Families Reveals Disruption of Putative Noncoding Regulatory DNA. Am. J. Hum. Genet. *98*, 58–74. https://doi.org/10.1016/J.AJHG.2015.11.023.
19. Leppa, V.M., Kravitz, S.N., Martin, C.L., Andrieux, J., Le Caignec, C., Martin-Coignard, D., DyBuncio, C., Sanders, S.J., Lowe, J.K., Cantor, R.M., and Geschwind, D.H. (2016). Rare Inherited and De Novo CNVs Reveal Complex Contributions to ASD Risk in Multiplex Families. Am. J. Hum. Genet. *99*, 540–554. https://doi.org/10.1016/j.ajhg.2016.06.036.
20. Marshall, C.R., Howrigan, D.P., Merico, D., Thiruvahindrapuram, B., Wu, W., Greer, D.S., Antaki, D., Shetty, A., Holmans, P.A., Pinto, D., et al. (2017). Contribution of copy number variants to schizophrenia from a genome-wide study of 41,321 subjects. Nat. Genet. *49*, 27–35. https://doi.org/10.1038/NG.3725.
21. Wilfert, A.B., Sulovari, A., Turner, T.N., Coe, B.P., and Eichler, E.E. (2017). Recurrent de novo mutations in neurodevelopmental disorders: properties and clinical implications. Genome Med. *9*, 101. https://doi.org/10.1186/S13073-017-0498-X.
22. Coe, B.P., Witherspoon, K., Rosenfeld, J.A., van Bon, B.W.M., Vulto-Van Silfhout, A.T., Bosco, P., Friend, K.L., Baker, C., Buono, S., Vissers, L.E.L.M., et al. (2014). Refining analyses of copy number variation identifies specific genes associated with developmental delay. Nat. Genet. *46*, 1063–1071. https://doi.org/10.1038/NG.3092.
23. de Bruijn, S.E., Fiorentino, A., Ottaviani, D., Fanucchi, S., Melo, U.S., Corral-Serrano, J.C., Mulders, T., Georgiou, M., Rivolta, C., Pontikos, N., et al. (2020). Structural Variants Create New Topological-Associated Domains and Ectopic Retinal Enhancer-Gene Contact in Dominant Retinitis Pigmentosa. Am. J. Hum. Genet. *107*, 802–814. https://doi.org/10.1016/J.AJHG.2020.09.002.
24. Cherry, T.J., Yang, M.G., Harmin, D.A., Tao, P., Timms, A.E., Bauwens, M., Allikmets, R., Jones, E.M., Chen, R., De Baere, E., and Greenberg, M.E. (2020). Mapping the *cis* -regulatory architecture of the human retina reveals noncoding genetic

variation in disease. Proc. Natl. Acad. Sci. USA *117*, 9001–9012. https://doi.org/10.1073/pnas.1922501117.

25. Van de Sompele, S., Small, K.W., Cicekdal, M.B., Soriano, V.L., D'haene, E., Shaya, F.S., Agemy, S., Van der Snickt, T., Rey, A.D., Rosseel, T., et al. (2022). Multi-omics approach dissects cis-regulatory mechanisms underlying North Carolina macular dystrophy, a retinal enhanceropathy. Am. J. Hum. Genet. *109*, 2029–2048. https://doi.org/10.1016/j.ajhg.2022.09.013.
26. Schwarz, N., Carr, A.-J., Lane, A., Moeller, F., Chen, L.L., Aguilà, M., Nommiste, B., Muthiah, M.N., Kanuga, N., Wolfrum, U., et al. (2015). Translational read-through of the RP2 Arg120stop mutation in patient iPSC-derived retinal pigment epithelium cells. Hum. Mol. Genet. *24*, 972–986. https://doi.org/10.1093/hmg/ddu509.
27. Schwarz, N., Lane, A., Jovanovic, K., Parfitt, D.A., Aguila, M., Thompson, C.L., da Cruz, L., Coffey, P.J., Chapple, J.P., Hardcastle, A.J., and Cheetham, M.E. (2017). Arl3 and RP2 regulate the trafficking of ciliary tip kinesins. Hum. Mol. Genet. *26*, 2480–2492. https://doi.org/10.1093/hmg/ddx143.
28. Nakano, T., Ando, S., Takata, N., Kawada, M., Muguruma, K., Sekiguchi, K., Saito, K., Yonemura, S., Eiraku, M., and Sasai, Y. (2012). Self-Formation of Optic Cups and Storable Stratified Neural Retina from Human ESCs. Cell Stem Cell *10*, 771–785. https://doi.org/10.1016/j.stem.2012.05.009.
29. Lane, A., Jovanovic, K., Shortall, C., Ottaviani, D., Panes, A.B., Schwarz, N., Guarascio, R., Hayes, M.J., Palfi, A., Chadderton, N., et al. (2020). Modeling and Rescue of RP2 Retinitis Pigmentosa Using iPSC-Derived Retinal Organoids. Stem Cell Rep. *15*, 67–79. https://doi.org/10.1016/j.stemcr.2020.05.007.
30. Patro, R., Duggal, G., Love, M.I., Irizarry, R.A., and Kingsford, C. (2017). Salmon provides fast and bias-aware quantification of transcript expression. Nat. Methods *14*, 417–419. https://doi.org/10.1038/nmeth.4197.
31. Frankish, A., Diekhans, M., Ferreira, A.-M., Johnson, R., Jungreis, I., Loveland, J., Mudge, J.M., Sisu, C., Wright, J., Armstrong, J., et al. (2019). GENCODE reference annotation for the human and mouse genomes. Nucleic Acids Res. *47*, D766–D773. https://doi.org/10.1093/nar/gky955.
32. Love, M.I., Huber, W., and Anders, S. (2014). Moderated estimation of fold change and dispersion for RNA-seq data with DESeq2. Genome Biol. *15*, 550. https://doi.org/10.1186/s13059-014-0550-8.
33. Love, M.I., Soneson, C., Hickey, P.F., Johnson, L.K., Pierce, N.T., Shepherd, L., Morgan, M., and Patro, R. (2020). Tximeta: Reference sequence checksums for provenance identification in RNA-seq. PLoS Comput. Biol. *16*, e1007664. https://doi.org/10.1371/journal.pcbi.1007664.
34. Ignatiadis, N., Klaus, B., Zaugg, J.B., and Huber, W. (2016). Data-driven hypothesis weighting increases detection power in genome-scale multiple testing. Nat. Methods *13*, 577–580. https://doi.org/10.1038/nmeth.3885.
35. Zhu, A., Ibrahim, J.G., and Love, M.I. (2019). Heavy-tailed prior distributions for sequence count data: removing the noise and preserving large differences. Bioinformatics *35*, 2084–2092. https://doi.org/10.1093/bioinformatics/bty895.
36. Rao, S.S.P., Huntley, M.H., Durand, N.C., Stamenova, E.K., Bochkov, I.D., Robinson, J.T., Sanborn, A.L., Machol, I., Omer, A.D., Lander, E.S., and Aiden, E.L. (2014). A 3D Map of the Human Genome at Kilobase Resolution Reveals Principles of Chromatin Looping. Cell *159*, 1665–1680. https://doi.org/10.1016/J.CELL.2014.11.021.
37. Melo, U.S., Schöpflin, R., Acuna-Hidalgo, R., Mensah, M.A., Fischer-Zirnsak, B., Holtgrewe, M., Klever, M.K., Türkmen, S., Heinrich, V., Pluym, I.D., et al. (2020). Hi-C Identifies Complex Genomic Rearrangements and TAD-Shuffling in Developmental Diseases. Am. J. Hum. Genet. *106*, 872–884. https://doi.org/10.1016/J.AJHG.2020.04.016.
38. Durand, N.C., Shamim, M.S., Machol, I., Rao, S.S.P., Huntley, M.H., Lander, E.S., and Aiden, E.L. (2016). Juicer Provides a One-Click System for Analyzing Loop-Resolution Hi-C Experiments. Cell Syst. *3*, 95–98. https://doi.org/10.1016/j.cels.2016.07.002.
39. Huang, H.-Y., Lin, Y.-C.-D., Cui, S., Huang, Y., Tang, Y., Xu, J., Bao, J., Li, Y., Wen, J., Zuo, H., et al. (2022). miRTarBase update 2022: an informative resource for experimentally validated miRNA–target interactions. Nucleic Acids Res. *50*, D222–D230. https://doi.org/10.1093/nar/gkab1079.
40. Ottaviani, D., LeCain, M., and Sheer, D. (2014). The role of microhomology in genomic structural variation. Trends Genet. *30*, 85–94. https://doi.org/10.1016/j.tig.2014.01.001.
41. Figuera, L.E., Pandolfo, M., Dunne, P.W., Cantú, J.M., and Patel, P.I. (1995). Mapping of the congenital generalized hypertrichosis locus to chromosome Xq24-q27.1. Nat. Genet. *10*, 202–207. https://doi.org/10.1038/ng0695-202.
42. Brewer, M.H., Chaudhry, R., Qi, J., Kidambi, A., Drew, A.P., Menezes, M.P., Ryan, M.M., Farrar, M.A., Mowat, D., Subramanian, G.M., et al. (2016). Whole Genome Sequencing Identifies a 78 kb Insertion from Chromosome 8 as the Cause of Charcot-Marie-Tooth Neuropathy CMTX3. PLoS Genet. *12*, e1006177. https://doi.org/10.1371/journal.pgen.1006177.
43. Zhu, H., Shang, D., Sun, M., Choi, S., Liu, Q., Hao, J., Figuera, L.E., Zhang, F., Choy, K.W., Ao, Y., et al. (2011). X-linked congenital hypertrichosis syndrome is associated with interchromosomal insertions mediated by a human-specific palindrome near SOX3. Am. J. Hum. Genet. *88*, 819–826. https://doi.org/10.1016/J.AJHG.2011.05.004.
44. DeStefano, G.M., Fantauzzo, K.A., Petukhova, L., Kurban, M., Tadin-Strapps, M., Levy, B., Warburton, D., Cirulli, E.T., Han, Y., Sun, X., et al. (2013). Position effect on FGF13 associated with X-linked congenital generalized hypertrichosis. Proc. Natl. Acad. Sci. USA *110*, 7790–7795. https://doi.org/10.1073/pnas.1216412110.
45. Bunyan, D.J., Robinson, D.O., Tyers, A.G., Huang, S., Maloney, V.K., Grand, F.H., Ennis, S., Silva, S.R.d., Crolla, J.A., and Mcmullan, T.F.W. (2014). X-Linked Dominant Congenital Ptosis Cosegregating with an Interstitial Insertion of a Chromosome 1p21.3 Fragment into a Quasipalindromic Sequence in Xq27.1. Open J. Genet. *4*, 415–425. https://doi.org/10.4236/ojgen.2014.46039.
46. Bowl, M.R., Nesbit, M.A., Harding, B., Levy, E., Jefferson, A., Volpi, E., Rizzoti, K., Lovell-Badge, R., Schlessinger, D., Whyte, M.P., and Thakker, R.V. (2005). An interstitial deletion-insertion involving chromosomes 2p25.3 and Xq27.1, near SOX3, causes X-linked recessive hypoparathyroidism. J. Clin. Invest. *115*, 2822–2831. https://doi.org/10.1172/JCI24156.
47. Haines, B., Hughes, J., Corbett, M., Shaw, M., Innes, J., Patel, L., Gecz, J., Clayton-Smith, J., and Thomas, P. (2015). Interchromosomal insertional translocation at Xq26.3 alters SOX3 expression in an individual with XX male sex reversal. J. Clin. Endocrinol. Metab. *100*, E815–E820. https://doi.org/10.1210/JC.2014-4383.

48. Boschann, F., Moreno, D.A., Mensah, M.A., Sczakiel, H.L., Skipalova, K., Holtgrewe, M., Mundlos, S., and Fischer-Zirnsak, B. (2022). Xq27.1 palindrome mediated interchromosomal insertion likely causes familial congenital bilateral laryngeal abductor paralysis (Plott syndrome). J. Hum. Genet. *67*, 405–410. https://doi.org/10.1038/S10038-022-01018-Z.
49. Middelkamp, S., Vlaar, J.M., Giltay, J., Korzelius, J., Besselink, N., Boymans, S., Janssen, R., De La Fonteijne, L., Van Binsbergen, E., Van Roosmalen, M.J., et al. (2019). Prioritization of genes driving congenital phenotypes of patients with de novo genomic structural variants. Genome Med. *11*, 1–15. https://doi.org/10.1186/S13073-019-0692-0/FIGURES/4.
50. Si, N., Meng, X., Zhao, Z., Xia, W., and Zhang, X. (2019). A 105 kb interstitial insertion in the Xq27.1 palindrome from pseudoautosomal region PAR1 causes a novel X-linked recessive compound phenotype. J. Transl. Med. *17*, 138. https://doi.org/10.1186/S12967-019-1887-2.
51. Dee, C.T., Hirst, C.S., Shih, Y.-H., Tripathi, V.B., Patient, R.K., and Scotting, P.J. (2008). Sox3 regulates both neural fate and differentiation in the zebrafish ectoderm. Dev. Biol. *320*, 289–301. https://doi.org/10.1016/j.ydbio.2008.05.542.
52. Helle, J.R., Barøy, T., Misceo, D., Braaten, Ø., Fannemel, M., and Frengen, E. (2013). Hyperphagia, mild developmental delay but apparently no structural brain anomalies in a boy without SOX3 expression. Am. J. Med. Genet. *161A*, 1137–1142. https://doi.org/10.1002/ajmg.a.35823.
53. Alatzoglou, K.S., Azriyanti, A., Rogers, N., Ryan, F., Curry, N., Noakes, C., Bignell, P., Hall, G.W., Littooij, A.S., Saunders, D., et al. (2014). SOX3 deletion in mouse and human is associated with persistence of the craniopharyngeal canal. J. Clin. Endocrinol. Metab. *99*, E2702–E2708. https://doi.org/10.1210/jc.2014-1160.
54. Jourdy, Y., Chatron, N., Carage, M.-L., Fretigny, M., Meunier, S., Zawadzki, C., Gay, V., Negrier, C., Sanlaville, D., and Vinciguerra, C. (2016). Study of six patients with complete F9 deletion characterized by cytogenetic microarray: role of the SOX3 gene in intellectual disability. J. Thromb. Haemost. *14*, 1988–1993. https://doi.org/10.1111/jth.13430.
55. Venceslá, A., Barceló, M.J., Baena, M., Quintana, M., Baiget, M., and Tizzano, E.F. (2007). Marker and real-time quantitative analyses to confirm hemophilia B carrier diagnosis of a complete deletion of the F9 gene. Haematologica *92*, 1583–1584. https://doi.org/10.3324/haematol.10693.
56. Barrett, S.P., Parker, K.R., Horn, C., Mata, M., and Salzman, J. (2017). ciRS-7 exonic sequence is embedded in a long noncoding RNA locus. PLoS Genet. *13*, e1007114. https://doi.org/10.1371/JOURNAL.PGEN.1007114.
57. Piwecka, M., Glažar, P., Hernandez-Miranda, L.R., Memczak, S., Wolf, S.A., Rybak-Wolf, A., Filipchyk, A., Klironomos, F., Jara, C.A.C., Fenske, P., et al. (2017). Loss of a mammalian circular RNA locus causes miRNA deregulation and affects brain function. Science *357*, eaam8526. https://doi.org/10.1126/SCIENCE.AAM8526.
58. Hanniford, D., Ulloa-Morales, A., Karz, A., Berzoti-Coelho, M.G., Moubarak, R.S., Sánchez-Sendra, B., Kloetgen, A., Davalos, V., Imig, J., Wu, P., et al. (2020). Epigenetic Silencing of CDR1as Drives IGF2BP3-Mediated Melanoma Invasion and Metastasis. Cancer Cell *37*, 55–70.e15. https://doi.org/10.1016/j.ccell.2019.12.007.
59. Hansen, T.B., Jensen, T.I., Clausen, B.H., Bramsen, J.B., Finsen, B., Damgaard, C.K., and Kjems, J. (2013). Natural RNA circles function as efficient microRNA sponges. Nature *495*, 384–388. https://doi.org/10.1038/NATURE11993.
60. Memczak, S., Jens, M., Elefsinioti, A., Torti, F., Krueger, J., Rybak, A., Maier, L., Mackowiak, S.D., Gregersen, L.H., Munschauer, M., et al. (2013). Circular RNAs are a large class of animal RNAs with regulatory potency. Nature *495*, 333–338. https://doi.org/10.1038/NATURE11928.
61. Cerda-Jara, C.A., Kim, S.J., Thomas, G., Farsi, Z., Zolotarov, G., Dube, G., Deter, A., Bahry, E., Georgii, E., Woehler, A., et al. (2024). miR-7 controls glutamatergic transmission and neuronal connectivity in a Cdr1as-dependent manner. EMBO Rep. *25*, 3008–3039. https://doi.org/10.1038/s44319-024-00168-9.
62. Liu, L., Liu, F.B., Huang, M., Xie, K., Xie, Q.S., Liu, C.H., Shen, M.J., and Huang, Q. (2019). Circular RNA ciRS-7 promotes the proliferation and metastasis of pancreatic cancer by regulating miR-7-mediated EGFR/STAT3 signaling pathway. Hepatobiliary Pancreat. Dis. Int. *18*, 580–586. https://doi.org/10.1016/J.HBPD.2019.03.003.
63. Ma, C., Gu, R., Wang, X., He, S., Bai, J., Zhang, L., Zhang, J., Li, Q., Qu, L., Xin, W., et al. (2020). circRNA CDR1as Promotes Pulmonary Artery Smooth Muscle Cell Calcification by Upregulating CAMK2D and CNN3 via Sponging miR-7-5p. Mol. Ther. Nucleic Acids *22*, 530–541. https://doi.org/10.1016/J.OMTN.2020.09.018.
64. Taylor, J.C., Martin, H.C., Lise, S., Broxholme, J., Cazier, J.B., Rimmer, A., Kanapin, A., Lunter, G., Fiddy, S., Allan, C., et al. (2015). Factors influencing success of clinical genome sequencing across a broad spectrum of disorders. Nat. Genet. *47*, 717–726. https://doi.org/10.1038/NG.3304.
65. Taliaferro, J.M., Aspden, J.L., Bradley, T., Marwha, D., Blanchette, M., and Rio, D.C. (2013). Two new and distinct roles for Drosophila Argonaute-2 in the nucleus: alternative pre-mRNA splicing and transcriptional repression. Genes Dev. *27*, 378–389. https://doi.org/10.1101/gad.210708.112.
66. Gao, F.-B. (2010). Context-dependent functions of specific microRNAs in neuronal development. Neural Dev. *5*, 25. https://doi.org/10.1186/1749-8104-5-25.

The American Journal of Human Genetics, Volume *112*

Supplemental information

Inter-chromosomal insertions at Xq27.1 associated with retinal dystrophy induce dysregulation of *LINC00632* and *CDR1as/ciRS-7*

Jessica C. Gardner, Katarina Jovanovic, Daniele Ottaviani, Uirá Souto Melo, Joshua Jackson, Rosellina Guarascio, Kalliopi Ziaka, Kwan-Leong Hau, Amelia Lane, Rachel L. Taylor, Niuzheng Chai, Christina Gkertsou, Owen Fernando, Monika Piwecka, Michalis Georgiou, Stefan Mundlos, Graeme C. Black, Anthony T. Moore, Michel Michaelides, Michael E. Cheetham, and Alison J. Hardcastle

Supplemental Figures

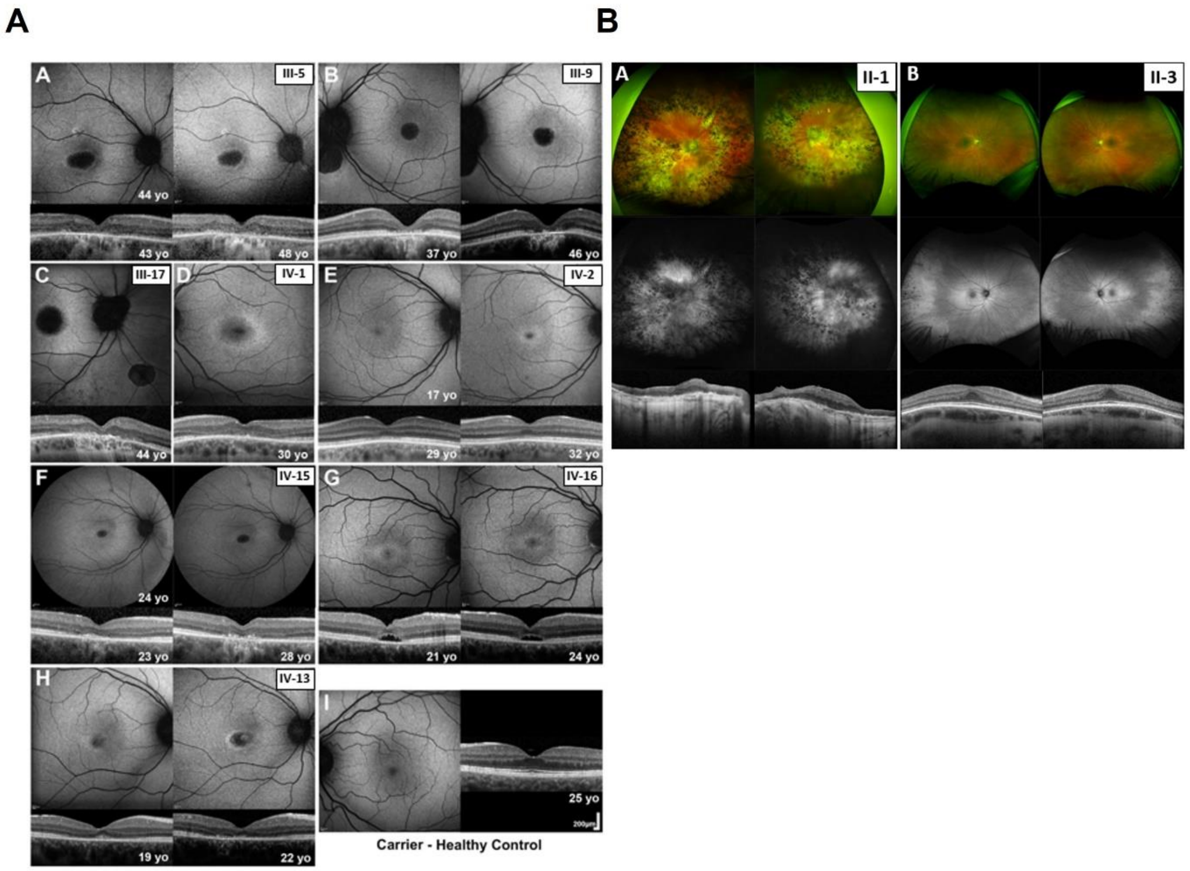

Figure S1: Retinal Imaging

(A) IRDF-1. (A-H) Fundus autofluorescence (FAF) and optical coherence tomography (OCT) of seven individuals with variable degrees of retinal degeneration. (I) FAF and OCT of an asymptomatic carrier as a healthy control. The age of each individual is indicated on the figure. For A, B, E, F, G, H, longitudinal data are presented showing progressive changes. For individuals C and D cross-sectional data are presented. The degeneration followed a cone-dystrophy/cone-rod dystrophy pattern, ranging from (E) a nearly normal FAF pattern and mild ellipsoid zone disruption on OCT to (C) advanced macular atrophy with decrease signal on FAF and atrophic changes on OCT. yo; years old. **(B)** IRDF-2. Top row colour fundus photographs, middle row FAF and bottom row transfoveal OCT of two subjects from IRDF-2. (A) Individual II:1 shows advanced atrophic changes. (B) Individual II:3 has a less severe phenotype with preserved foveal structure, tapetal like reflex, and peripheral degenerative changes.

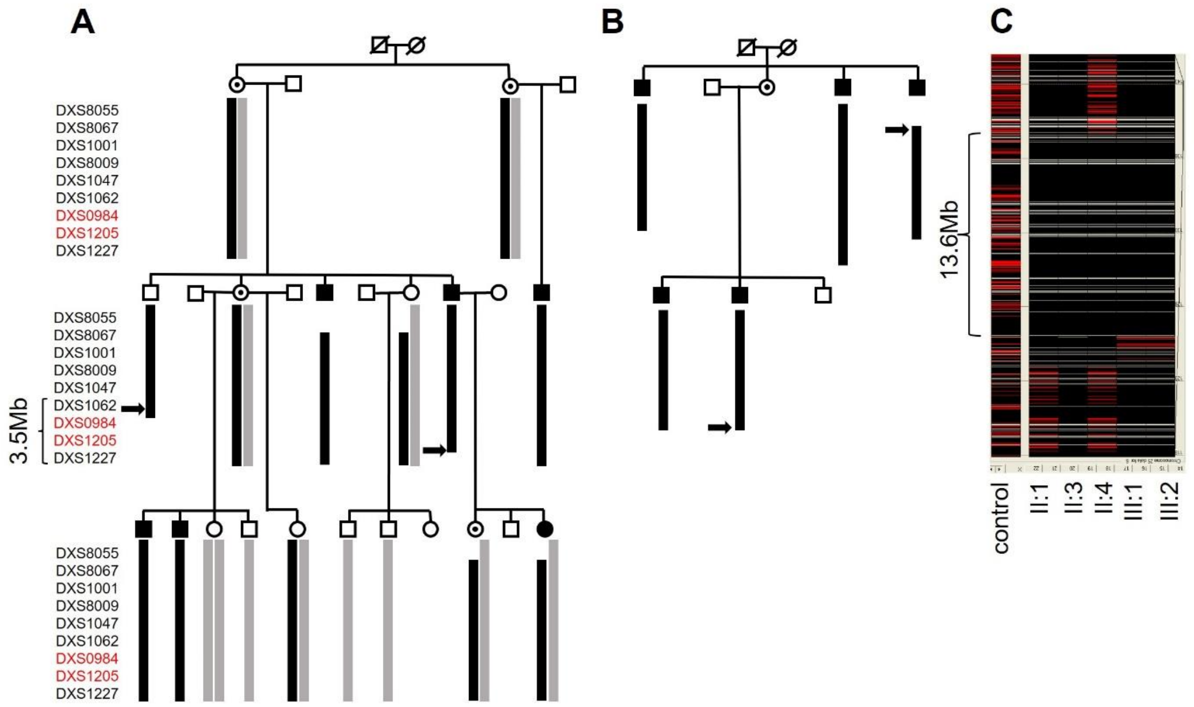

Figure S2: Haplotype analysis

(A) IRDF-1. Black bars indicate chromosome X retinal dystrophy associated haplotypes. Gray bars indicate X chromosome haplotypes that do not segregate with disease. Arrows represent individuals with informative crossovers that define a 3.5Mb retinal dystrophy shared haplotype (chrX:138,221,084-141,714,225 hg38) on Xq26.3-Xq27.2. (markers linked with retinal dystrophy highlighted in red). **(B)** IRDF-2. Black bars indicate chromosome X retinal dystrophy haplotypes in five affected individuals. **(C)** Chromosome X SNP array genotyping data of 5 affected individuals showing a 13.6Mb linked region on Xq25-21.1 in black (chrX:126,856,362-140,533,499 hg38). An unaffected control sample is shown on the left.

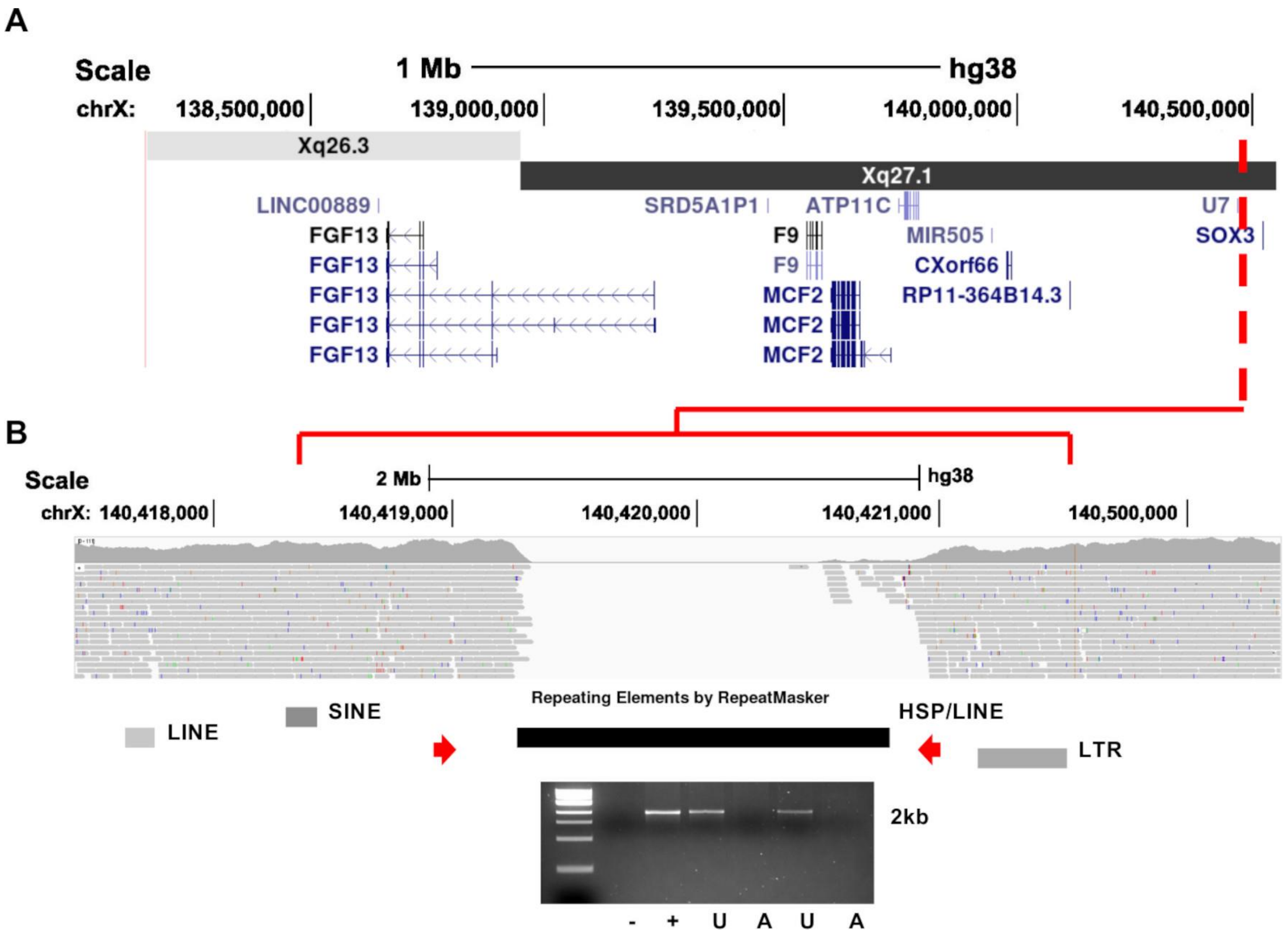

Figure S3: Identification of a genomic region on Xq27.1 poorly covered by NGS

(A) Genomic location (dotted red line) of the sequence gap 80kb downstream of *SOX3* in NGS data for IRDF-1 on Xq27.1. **(B)** Enlarged IGV alignment of NGS gap across a human specific palindrome (HSP) on Xq27.1 (black bar) flanked by an upstream short interspersed nuclear element (SINE) and a downstream long tandem repeat (LTR). Agarose gel (bottom) showing PCR amplification across the gap with primers flanking the HSP (arrows). A 2kb amplicon is present in control (+) and unaffected (U) IRDF-1 individuals, but not in affected individuals (A), indicating a possible SV.

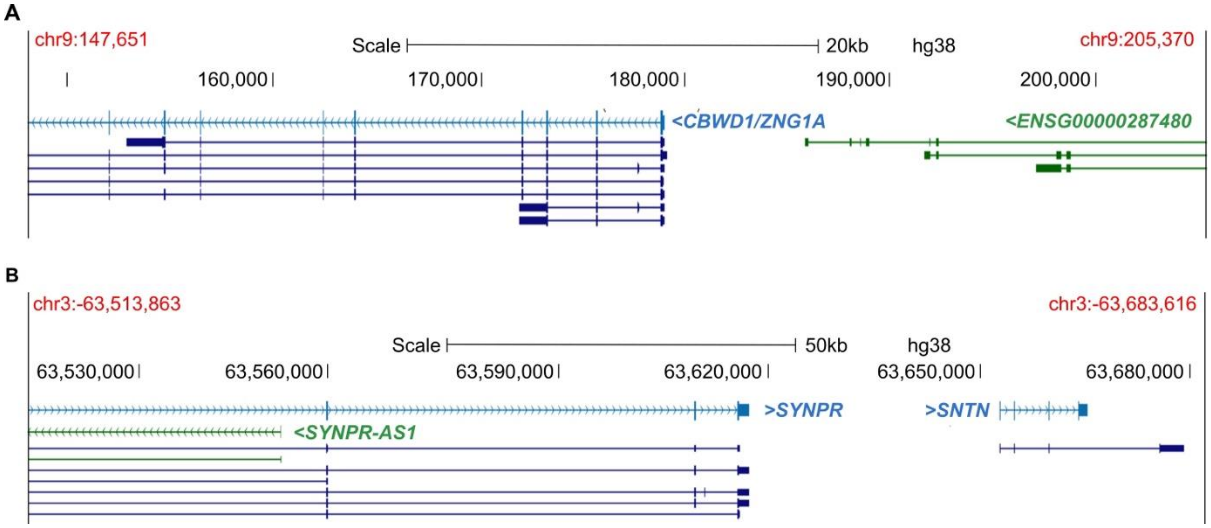

Figure S4: Genes and transcripts within the 9q24.3 and 3p14.2 inter-chromosomal insertions
(A) A 58kb 9p24.3 inter-chromosomal insertion identified in family IRDF-1 contains exons 1-10 and the upstream region of the *CBWD1/ZNG1A* gene and terminal exons of a non-coding RNA. **(B)** A 169kb inverted inter-chromosomal insertion identified in family IRDF-2 contains the *SNTN* gene, three terminal exons of *SYNPR* and the upstream region and first exon of *SYNPR-AS1*. Breakpoint (BP) co-ordinates (in red) for the insertions are also shown.

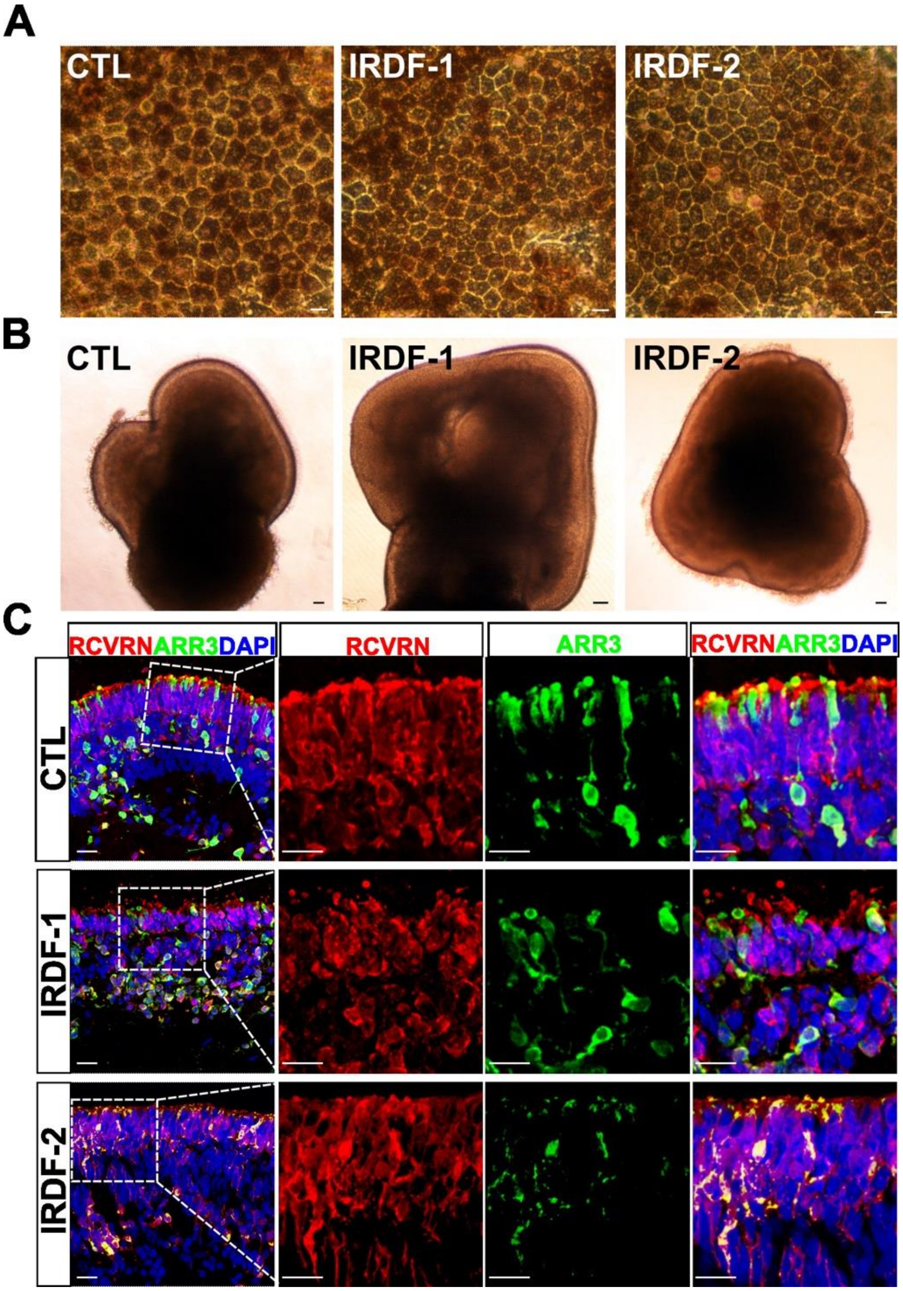

Figure S5: Characterisation of IRDF-1, IRDF-2 and control iPSC-derived RPE and retinal organoids

(A) Brightfield microscopy images of RPE derived from control (CTL), IRDF-1 and IRDF-2 derived iPSC, as indicated. Note the hexagonal shape and pigmentation characteristic of RPE. Scale bar 20 µm**. (B)** Brightfield microscopy images of representative retinal organoids derived from CTL, IRDF-1 and IRDF-2 iPSC, as indicated. Scale bar 50 µm. **(C)** Immunohistochemistry of 10 µm cryosections of D150 retinal organoids from CTL, IRDF-1 and IRDF-2 iPSC, as indicated, stained with primary antibodies against photoreceptor markers recoverin (RCVRN, red), cone arrestin (ARR3, green) showing rod and cone photoreceptor differentiation. Nuclei are stained with DAPI (blue), note the discrete outer nuclear layer of photoreceptors confirming retinal organoid lamination. Scale bar 20 µm.

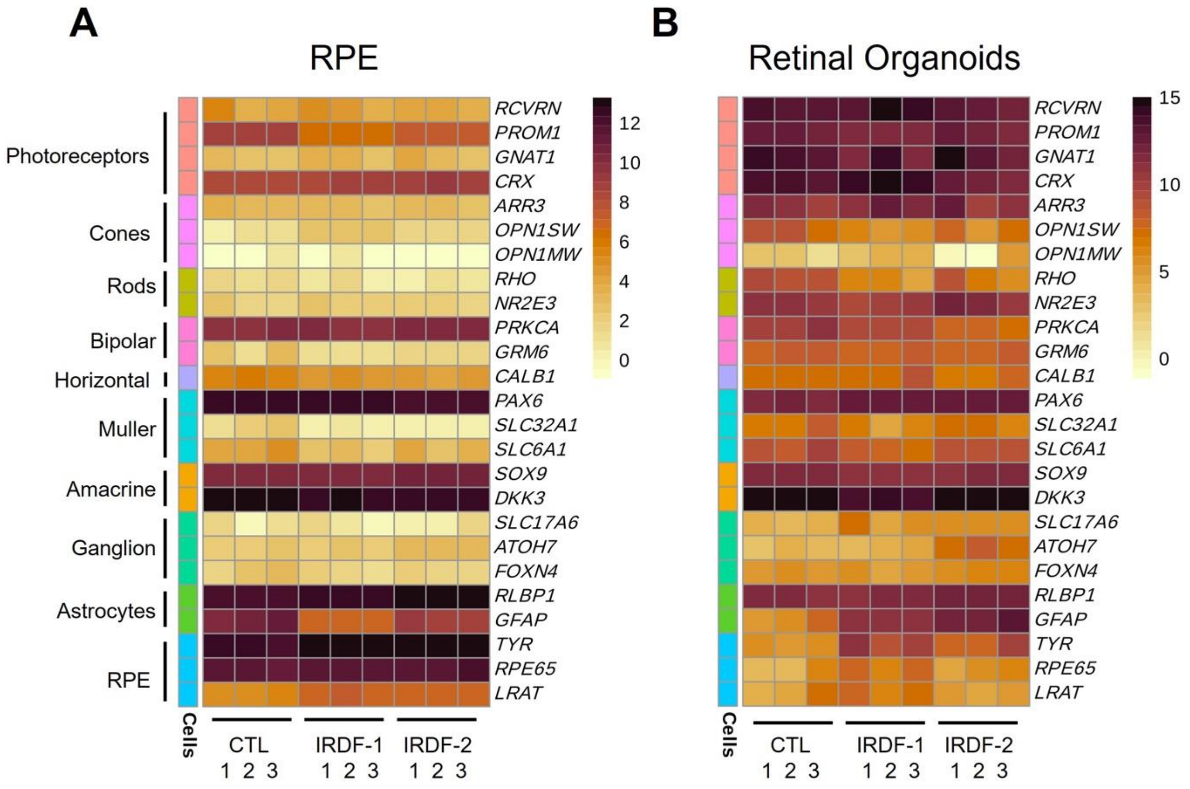

Figure S6: Heat maps of gene expression profiles generated from RNA-seq data from RPE and retinal organoids

(A) Gene profiles of RPE samples (3 samples per line). **(B)** Marker gene profiles of D150 ROs retinal cell types including photoreceptor-specific genes. Comparison between control (CTL) and IRDF-1 and IRDF-2 (3 ROs per line). Both tissue types differentiated successfully and expressed tissue specific genes.

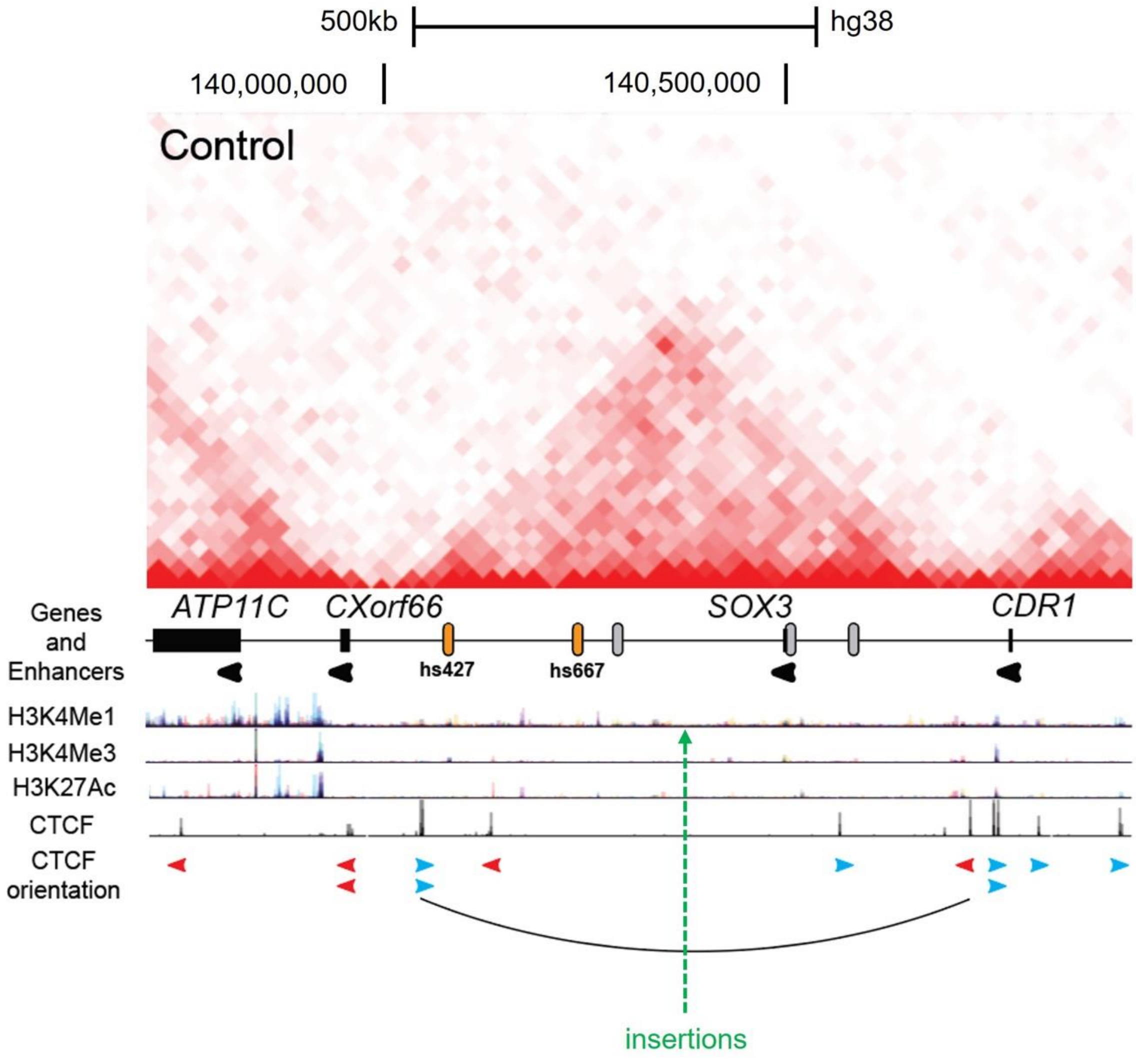

Figure S7: CTCF-associated *SOX3* TAD boundaries

CTCFs at the *SOX3* TAD boundaries are arranged in the classical convergent orientation. The position of the insertions is indicated by the green arrow.

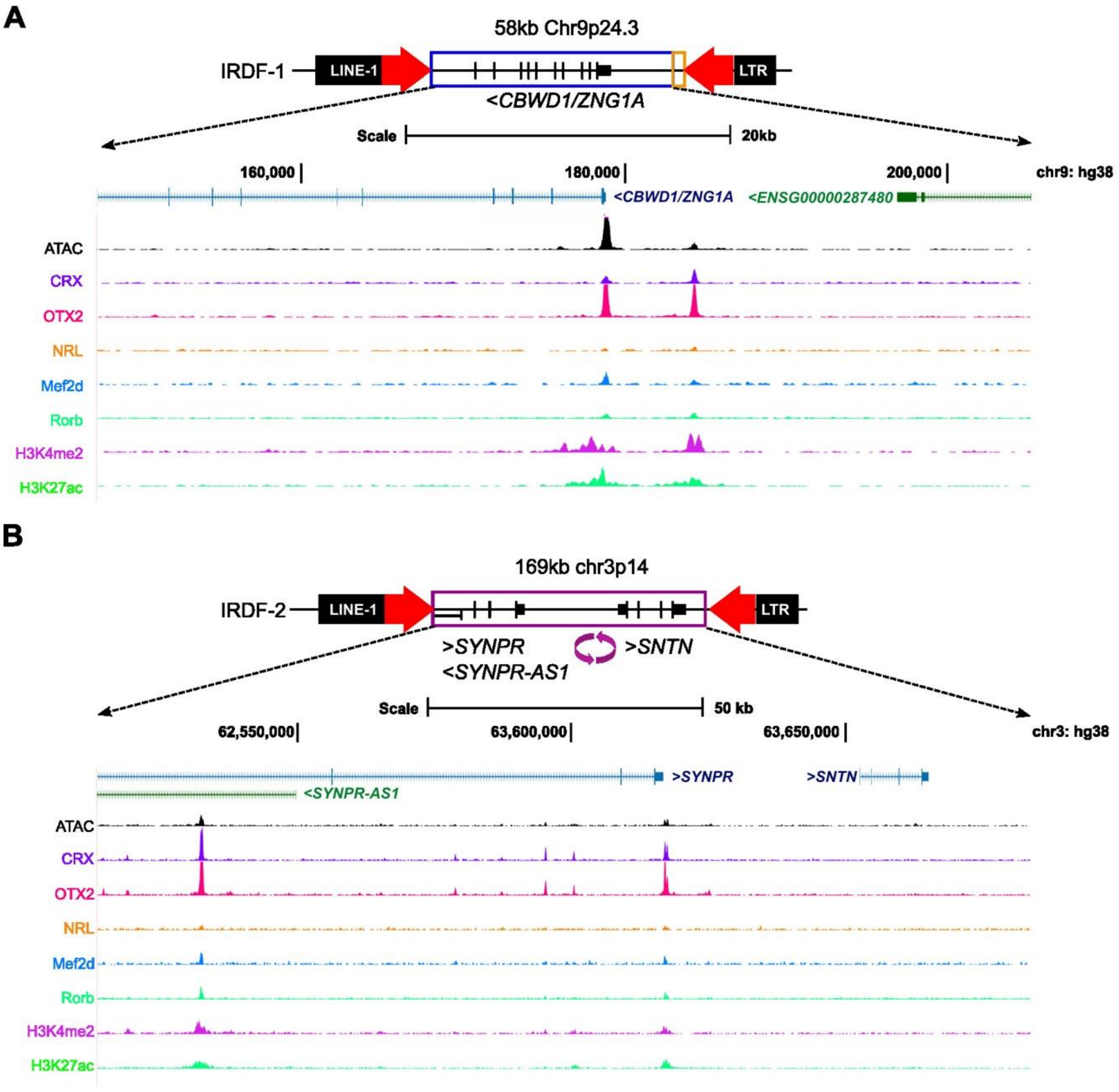

Figure S8. Active regulatory elements correspond with photoreceptor specific enhancers within the ICIs

Multiomic data aligned to **(A)** the 9p24.3 insertion in IRDF-1 and **(B)** the 3p14.2 insertion in IRDF-2 reveals open chromatin (ATAC, H3K27ac) and signatures of active retina specific enhancers (*OTX2*, *CRX*) within both insertions.

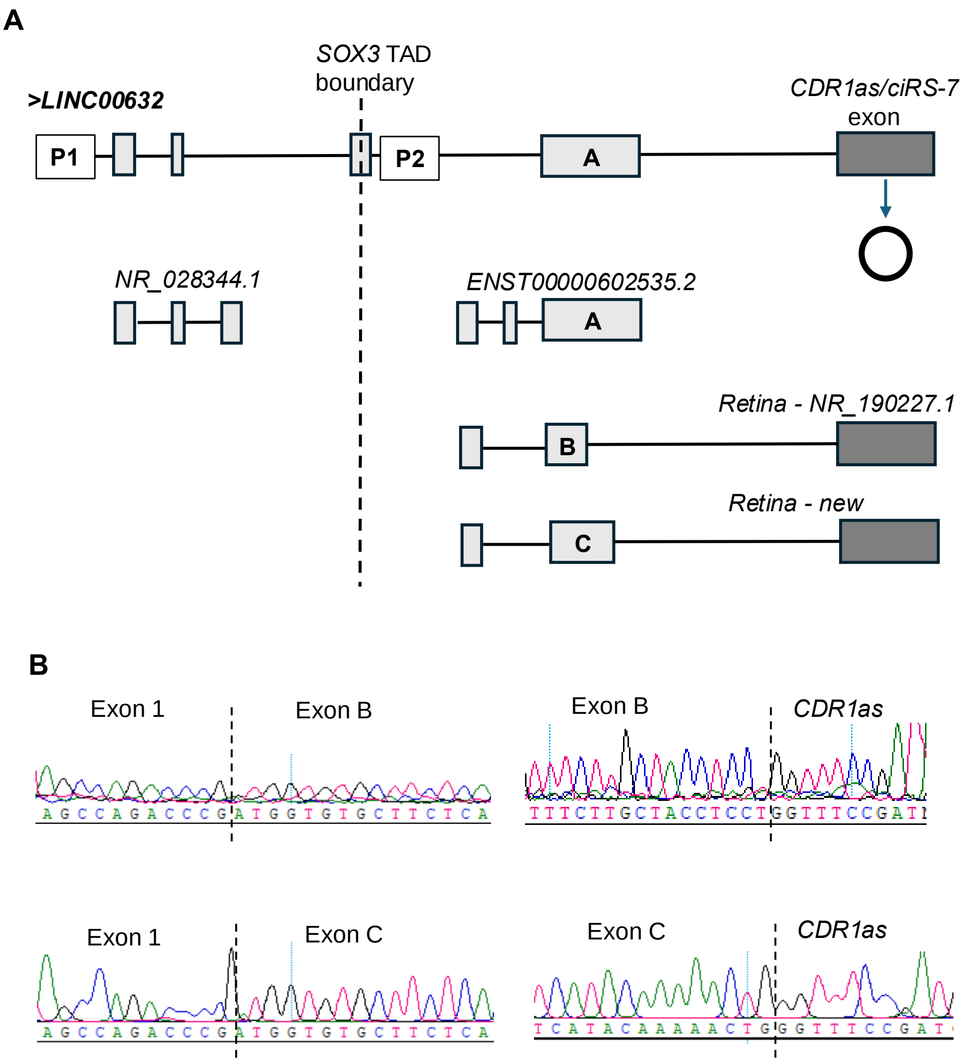

Figure S9: Expression of *CDR1as/ciRS-7* in retina is driven by two promoters on different sides of the *SOX3* TAD boundary

(A) Schematic showing *LINC00632* transcripts and promoters, P1 and P2, relative to the position of the distal *SOX3* TAD boundary. **(B)** Sequence chromatograms of two shorter transcripts with a terminal *CDR1as* exon detected by RT-PCR in D150 control retinal organoids showing the splice junctions. Both transcripts are associated with P2 and lie outside of the *SOX3* TAD boundary. Note, not all putative *LINC00632* transcripts in ENSEMBL or UCSC are depicted on this figure.

Supplemental Tables

Table S2: Primers used for the identification and characterisation of SVs and to characterise linear *LINC00632* transcripts

Target	Primer Name	Primer sequence (5'-3')	Position hg38
**Human Specific Palindrome**	HSPF	CATTGAAATCAACCACCCAG	chrX:140,418,915
	HSPR	AACATTTCCAATCTCCCCAAC	chrX:140,420,906
**DNA Walking**	R-TSP1	GGTCCAATGCTTTGTAGTG	chrX:-140,421,014
	R-TSP2	ACCAGAGTTCTCTGTGCATTG	chrX:-140,420,964
	R-TSP3	AACATTTCCAATCTCCCCAAC	chrX:-140,420,906
	F-TSP1	GTAGTGTGATGCCTCCAGC	chrX:140,420,580
	F-TSP2	ATTGACTTGGCGATGCG	chrX:140,420,618
	F-TSP3	GAAGAAAGTCATTGGGGTTCAGC	chrX:140,420,686
**IRDF-1 BP**	Chr9R1	AAATATGCTCAGTAGCGGGG	chr9:-147723
***LINC00632* (P2) linear transcripts with promoter outside SOX3 TAD**	Forward<br>Reverse	AGCCAGACCCGATGGTGTG<br>AAGACCCGGAGTTGTTGGAA	Exon1/2<br>CDR1as
***LINC00632*/NR_19022.1 nested from *LINC00632* (P2)**	Forward<br>Reverse	AGCCAGACCCGATGGTGTG<br>ATCGGAAACCAGGAGGTAGC	Exon1/2<br>Ex2A/CDR1as
***LINC00632*/NEW nested from *LINC00632* (P2)**	Forward<br>Reverse	AGCCAGACCCGATGGTGTG<br>GTGCCATCGGAAACCCAG	Exon1/2<br>Ex2B/CDR1as

Table S4: RT-qPCR primers used to validate differential expression of linear and circular isoforms of *LINC00632*

Target	Primer	Primer sequence (5’-3’)
ACTIN	Forward	CCAACCGCGAGAAGATGA
	Reverse	CCAGAGGCGTACAGGGATAG
GAPDH	Forward	CCCCACCACACTGAATCTCC
	Reverse	GGTACTTTATTGATGGTACATGACAAG
LINC00632-NR_028344.1	Forward	AGACAGCATGCCACTGGAAA
	Reverse	CTGCGGACACAAGTCTGCTT
CDR1as/ciRS-7	Forward	CGTCTCCAGTGTGCTGATCT
	Reverse	AAGACCCGGAGTTGTTGGAA

Supplemental Methods

Genotyping

The IRDF-1 locus was initially established using polymorphic markers on ChrX that were genotyped in 17 individuals (ABI PRISM® linkage mapping set version 2.5, Thermo Fisher Scientific, UK; and additional microsatellite markers - primers and conditions available upon request). The locus was subsequently refined by SNP genotyping following genome sequence analysis of additional individuals (two affected IV-15, IV-16 and unaffected III-1). SNVs were validated using PCR amplification and Sanger Sequencing (primers and conditions available on request). The IRDF-2 locus was identified using SNP genotyping in 5 affected individuals. Genome-Wide SNP analysis was carried out using the Affymetrix Genome-Wide SNP6.0 microarray. Genotypes were generated using the Birdseed V2 algorithm with a confidence threshold of 0.01 and copy number data was generated using the SNP 6.0 CN/LOH Algorithm both within the Affymetrix Genotyping console. Autozygosity analysis was carried out using AutoSNPa (http://dna.leeds.ac.uk/autosnpa/). Copy number results were analysed using the Affymetrix Chromosome Analysis Suite.

Sanger sequencing of candidate genes

In IRDF-1 and IRDF-2 the coding regions and intron/exon boundaries of all candidate genes (including reference and predicted genes in GRCh37/hg19) within the linked loci were amplified by PCR and bidirectionally sequenced under standard conditions using the BigDye Terminator v 3.1 kit (Life Technologies).

Array Comparative Genome Hybridisation (CGH)

In IRDF-1, genomic DNA of an affected male (III-5) was analysed for copy number variations (CNVs) of the X chromosome by array CGH (Roche, NimbleGen, Madison, USA) using the NimbleGen custom microarray services facility (NimbleGen Systems of Iceland, LLC, Reykjavik, Iceland). DNA was labelled with Cy3 or Cy5 using a NimbleGen Dual Color DNA labelling kit and co-hybridized to the arrays (NimbleGen CGH Services: Guide to your CGH data v5p1). Design of the array was based on GRCh36/hg18.

Exome and genome sequencing

WES was performed for one affected individual of IRDF-1 (IV-1) and IRDF-2 (III-1). Library preparation was performed using the Agilent Human Exome V4 capture kit and run on an Illumina HiSeq2500TM system by Otogenetics Corporation (Atlanta, USA). After quality control, reads were aligned to the GRCh37 sequence with BWA v.0.78 and variants were called using GATK HaplotypeCaller V.3.3. CNVs and SVs were analysed from WES data using ExomeDepth, Manta Structural Variant Caller, Canvas Copy Number Variant Caller and Control-FREEC.

In IRDF-1 targeted genome sequencing of the linked region on chrX was performed in one affected male (III-5) and two unrelated male control samples. Genomic DNA was independently captured using a Sequence Capture 2.1M Custom Array (Roche, NimbleGen) to enrich five target regions of interest (chrX: 8,353,912–19,064,721; 37,980,770–38,109,739; 46,569,929–46,638,096; 135,964,421–144,028,731; 153,000,017–153,246,396; GRCh36/hg18). 100bp paired-end sequencing was performed on an Illumina Genome Analyzer II system (Source Bioscience Geneservice). Initial bioinformatics analysis was performed using CASAVA (Illumina). Advanced bioinformatic analysis was performed using the SAMtools software toolkit (SNP and DIP detection). SNPs were called with the MAQ alignment and downstream analysis tools (Source Bioscience Geneservice). Data were then interrogated using a variety of bioinformatics tools.

Short read WGS was performed using Macrogen Inc. (Seoul, Korea) on Illumina HiSeqX machines using a 2x 100bp paired end module with a minimum median coverage per genome of 30-fold. For IRDF-1, two affected individuals (IV-15, IV-16) and one unaffected (III-1) were sequenced. For IRDF-2, one affected individual (III-1) was sequenced. After quality control, reads were aligned with BWA to map reads against reference genomes (GRCh37/hg37 and GRCh38/hg38) and GATK HaplotypeCaller (V.3.3) for variant calling. Variants were filtered using gnomAD MAF<0.001, validated with IGV software (V.2.4), and selected for purposes of refining the locus. CNVs and SVs were analysed using Canvas Copy Number Variant Caller (Illumina) and Manta Structural Variant Caller.

Identification and validation of structural variants

A dark or camouflaged genome sequence gap within the IRDF-1 locus was investigated using long range PCR amplification, with sequence specific primers HSPF and HSPR (Table S2, PCR conditions available on request). The 1.8kb sequence gap spanned a complex intergenic repeat on Xq27.1, comprising a 180bp human specific palindrome, flanked by long tandem repeat (LTR) and a long intergenic nuclear element (LINE/L1PA3).

A genome walking strategy to identify unknown sequence within the gap was performed using genomic DNA from an affected male (IV-1) in IRDF-1 and was subsequently repeated in IRDF-2 (III-1). The DNA Walking SpeedUp™ premix kit (Seegene, Inc. Seoul, Korea) was used according to the manufacturer's instructions. gDNA from an affected individual was amplified using DW-ACP™ primers from the kit paired with a set of nested target-specific (TSP) primers designed against the centromeric (TSPF1-3) and telomeric (TSPR1-3) flanks of the palindrome (Table S2). An initial DNA walking PCR, using primers DW-ACP™ and TSP1, was employed to amplify unknown sequence, followed by two rounds of PCR using DW primers and nested TSP2-3 primers with products from the previous PCR. The amplification products were separated by agarose gel electrophoresis and extracted using a Qiaquick gel extraction DNA kit (Qiagen,UK) before Sanger sequencing. Breakpoint PCR was performed to validate the proximal chrX breakpoint of IRDF-1 using primers HSPF and Chr9R1 (Table S2). PCR conditions are available on request.

Interrogation of the genomic region

To explore the regulatory landscape of the chrX region and inter-chromosomal insertion regions on Chr9p and Chr3p we interrogated the UCSC encode data sets and the integrated retinal multi-omic data base RegRet.[1]

Cell culture

Fibroblasts were cultured in Dulbecco's Modified Eagle Medium (DMEM; Thermo Fisher Scientific) supplemented with 15% fetal bovine serum (FBS; Thermo Fisher Scientific), 1% L-glutamine (Thermo Fisher Scientific), and 1% penicillin-streptomycin (Thermo Fisher Scientific) before reprogramming to iPSC.

Preparation of Hi-C libraries

Hi-C libraries were processed as described previously.[2] In brief, ~1 million cells were fixed in 2% formaldehyde, lysed, and digested overnight with DpnII enzyme (New England BioLabs, R0543). Digested DNA ends were marked with biotin-14-dATP (Thermo Fisher Scientific, 19524016) and ligated overnight using T4 DNA ligase (New England BioLabs, M0202). Formaldehyde crosslinking was reversed by incubation in 5 M NaCl for 2 h at 68°C, followed by ethanol precipitation. Covaris (S-Series 220) was used to shear the DNA to fragments of 300–600bp for library preparation, and biotin-filled DNA fragments were pulled down using Dynabeads MyOne Streptavidin T1 beads (Thermo Fisher Scientific, 65602). The DNA ends were subsequently repaired using T4 DNA polymerase and the Klenow fragment of DNA polymerase I (New England BioLabs, M0203 and M0210) and phosphorylated with T4 Polynucleotide Kinase NK (New England BioLabs, M0201). The DNA was further prepared for sequencing by ligating adaptors to the DNA fragments, using the NEBNext Multiplex Oligos for Illumina kit (New England BioLabs, E7335 and E7500). Indexes were added via PCR amplification (4–8 cycles) using the NEBNext Ultra II Q5 Master Mix (New England BioLabs, M0544). PCR purification and size selection were carried out using Agencourt AMPure XP beads (Beckman Coulter, A63881). Libraries were deep sequenced (~240 million fragments for fibroblasts and 320 million fragments for retinal organoids) in a 75bp paired-end run on a HiSeq4000 (Illumina). For each line, the Hi-C library was created by pooling a total of four technical replicates to ensure higher complexity of the sequencing library.

References

1. Van de Sompele, S., Small, K.W., Cicekdal, M.B., Soriano, V.L., D'haene, E., Shaya, F.S., Agemy, S., Van der Snickt, T., Rey, A.D., Rosseel, T., et al. (2022). Multi-omics approach dissects cis-regulatory mechanisms underlying North Carolina macular dystrophy, a retinal enhanceropathy. Am J Hum Genet *109*, 2029–2048. https://doi.org/10.1016/j.ajhg.2022.09.013.
2. Melo, U.S., Schöpflin, R., Acuna-Hidalgo, R., Mensah, M.A., Fischer-Zirnsak, B., Holtgrewe, M., Klever, M.K., Türkmen, S., Heinrich, V., Pluym, I.D., et al. (2020). Hi-C Identifies Complex Genomic Rearrangements and TAD-Shuffling in Developmental Diseases. Am J Hum Genet *106*, 872–884. https://doi.org/10.1016/J.AJHG.2020.04.016.
